# Use of energy dispersive X-ray fluorescence to authenticate European wines with protected designation of origin. Challenges of a successful control system based on modelling

**DOI:** 10.1016/j.foodchem.2024.141989

**Published:** 2025-02-15

**Authors:** Michele Ghidotti, Sergej Papoci, Arantza Respaldiza, Håkan Emteborg, Franz Ulberth, María Beatriz de la Calle Guntiñas

**Affiliations:** European Commission, Joint Research Centre (JRC), Geel, Belgium

**Keywords:** Wine, PDO, EDXRF, Fraud, Elemental profile, Multivariate analysis

## Abstract

Consumers are willing to pay a higher price for food with geographical origin labels such as Protected Designation of Origin and Protected Geographical Indication. In this work, the elemental profile of wine obtained by XRF, combined with multivariate analyses, is used to authenticate 111 Croatian, Italian and Spanish red and white wines, 102 of them from 20 Protected Designations of Origin, reproducing the circumstances faced by control laboratories, using commercially available wines without traceability records. Wines that shared origin clustered together and separated from those of other regions following multivariate statistical tests. Classifications made using Soft Independent Modelling by Class Analogy were characterised by poor sensitivity and specificity. An alternative approach based on successive Partial Least Square Discriminant Analyses with consecutive classifications at country, region and finally, Protected Designation of Origin level, was developed and implemented with good accuracy results. In total, 88 % of the samples were correctly classified.

## Highlights

• Elemental profiles allow authentication of European wines with PDO claims.
• ED-XRF is a fast and clean approach to authenticate geographical origin in PDO wines.
• PLS-DA is more accurate than SIMCA in geographical authentication of PDO wines.
• International collaboration is needed to carry out PDO wine authentication controls.

## 1 Introduction

Wine is a product with high economic significance, not only in countries with well-recognised wine production culture, but also in emerging economies where wine is replacing other traditional products (Wu et al., 2019). Consumers are willing to pay high prices for wine, in particular for wines with quality labels such as the Protected Designation of Origin (PDO) and Protected Geographic Indication (PGI) granted by the European Union (*Regulation (EU*) 1151/2012 of the European Parliament and of the Council of 21 November 2012 on quality schemes for agricultural products and foodstuffs, 2012). There are more than thousand PDO wines registered in eAmbrosia, the EU geographical indication register. Economic profit is the main driver for food fraud and therefore, products with a PDO label are attractive targets for fraudsters.

The European Union Farm to Fork Strategy (Farm to Fork Strategy, 2024) states: “*Food fraud jeopardises the sustainability of food systems. It deceives consumers and prevents them from making informed choices. It undermines food safety, fair commercial practices, the resilience of food markets and ultimately the single market*”. Although the Official Controls Regulation (EU) 2017/625 (*Regulation (EU*) 2017/625 of the European Parliament and of the Council of 15 March 2017 on official controls and other official activities performed to ensure the application of food and feed law, rules on animal health and welfare, plant health and plant protection products, 2017) mandates competent authorities to carry out official controls to detect fraudulent or deceptive practices, there is a lack of fully validated methods to detect food fraud, which has a negative impact in the involvement of control laboratories. Up to now, traceability controls have been based on paper documentation. Frequently, authentication of geographical origin, botanical variety, and production agricultural system (organic/conventional), needs the support of multivariate analysis, artificial intelligence and modelling. Although some efforts have been done by international standardisation organisations such as Eurachem (EURACHEM, 2021) and AOAC international (Wehling et al., 2011), which have addressed the issue of validation of qualitative analytical methods, investment in validation protocols for prediction models need to be made.

Many articles have been published on the power of multi-elemental analyses, alone or in combination with other type of analyses such as stable isotope ratios (IR), strontium isotope ratios, and nuclear magnetic resonance (NMR), to authenticate the geographical origin of wine (Aceto et al., 2020; da Costa et al., 2020; Duley et al., 2021; Ghezzi et al., 2018; Orellana et al., 2019). A fraction of the elements in wine is endogenous and related to the type of soil where the vine grew, grape variety, maturity and climatic conditions, and another fraction is exogenous and can be related to the use of fertilisers and pesticides, environmental pollution, and wine making processes (Grindlay et al., 2011). Some studies carry out analyses of the soil where the grapes are grown, stablishing a link with the elemental profiles of the wines from that region (Aceto et al., 2020; Catarino et al., 2018).

In most studies in which authentication is based on the elemental composition of wine, analyses are carried out by Inductively Coupled Plasma (ICP) (Grindlay et al., 2011) coupled to Atomic Emission Spectrometry (AES) (Aceto et al., 2020) or to Mass Spectrometry (MS) (da Costa et al., 2020; Duley et al., 2021; Orellana et al., 2019). ICP-based methods are characterised by low limits of quantification (LOQ), at the sub-ppm level; however, the determination of certain elements such as Cl, and Br, that can be important for classification purposes, is difficult by those methods. Frequently, wine is diluted before measurement using diluted HNO_3_ (Ranaweera et al., 2021). Analyses based on the use of Energy Dispersive X-ray Fluorescence Spectroscopy (EDXRF), have the advantage that they do not require the use of harsh chemical reagents reducing the risk for the analyst, and both, the environmental impact, and the cost of the analyses. On the negative side, EDXRF-based methods are characterised by relatively high LOQs (low ppm level). The concentrations in wine of some elements such as rare earths, are too low to be detected by EDXRF; however, the determination of Cl and Br is straightforward with this technique. The technique has been successfully applied in our laboratory to authenticate the geographical origin of honey (Fiamegos et al., 2020; Ghidotti et al., 2021), paprika (Fiamegos et al., 2021), and rice (Dumitrascu et al., 2021).

Many of the studies published, demonstrate the feasibility of using a certain analytical approach in combination with multivariate analyses and modelling, to authenticate the geographical origin of wine produced with a single grape variety, belonging to one or two vintages (Duley et al., 2021; Ranaweera et al., 2021). However, few of those studies provide information on the performance of such predictive models on unknown/unlabelled samples. If authentication analyses are to be carried out in the frame of control analyses to implement legislation put in place to fight fraud, methods need to undergo a full validation, providing information about sensitivity (true positives, TP), specificity (true negatives, TN) and accuracy. Most of the studies that include method validation, analyse hundreds of samples, frequently from different countries (Wu et al., 2019) or even continents (Hu et al., 2019). However, how realistic it is that a control laboratory will have access to hundreds of wine samples from a particular region, possibly from the same botanical origin and vintage, and with traceability records, to build up the needed models? This question is particularly pertinent in the sector of PDO/PGI wines, considering the more than thousand registered European PDO labels and the attractiveness of those wines for counterfeiters. Hence, control laboratories have to take decisions on the authenticity of a wine sample without having access to reference values for authentic wines of a certain PDO/PGI.

This work has two objectives. The first is to propose a protocol that can be used by control laboratories for authentication of wines with PDO/PGI labels in the situation that they face at present, namely, lack of reference values for all the existing PDO/PGI wines on the European market. The protocol is based on the analyses of a relatively reduced number of wine samples purchased at retailers, mimicking a realistic scenario for control laboratories. The second is to test if the elemental profile of wine determined by EDXRF, in combination with multivariate analyses, can be used to authenticate the geographical origin of a wide variety of wines with PDO label. Limitations of the approach if it were to be used by control laboratories are discussed, as well as different alternatives to address those limitations.

The study was conducted on 102 Croatian, Italian and Spanish red and white wines with 20 different PDOs. Principal Component Analyses (PCA), Soft Independent Modelling by Class Analogy (SIMCA), cluster analyses, and Partial Least Square Discriminant Analyses (PLS-DA) are the techniques used for multivariate statistical analyses. EDXRF was used for the elemental characterisation of wine.

## 2 Methods

### 2.1 Samples

In this study, we tried to reproduce the scenario that a control laboratory would face when having to authenticate wine with a PDO label, possibly from a different country than that of the control laboratory. For this reason, only a relatively low number of commercially available wines (4 to 12 samples per PDO), were used as reference samples to build up models for authentication purposes.

One hundred and eleven wines were analysed in this study, all of them purchased at supermarkets and retailers, some of them specialised in wine. The study included 42 Croatian (19 red, 23 white), 26 Italian (all red) and 43 Spanish (32 red, 11 white) wines. The following PDOs, summarised in Table 1, were included in the study: Hrvatska Istra, Slavonija, Srednja i Južna Dalmacija, Plešivica, Dingač, Moslavina, Ponikve, Sjeverna Dalmacija, Zapadna Kontinentalna Hrvatska, Chianti, Chianti Classico, Morellino di Scansano, Bolgheri, Bierzo, Rioja, Ribera del Duero, Toro, Valdeorras, Catalunya, and La Mancha. The study includes also one red wine from the Castilla y León PGI, 8 red wines without PDO or PGI claims (6 Italian red wines from Toscana and 1 Spanish red wine from the region Castilla-La Mancha) and 1 Spanish white wine from the region Castilla y León. Several production years were covered, 2009, 2011, 2012, 2015, 2016, 2017, 2018, 2019, 2020 and 2021. Detailed information about the wines analysed in this study, including country and region of origin, PDO/PGI, grape variety and production year, is given as supplementary material, Supplementary 1. After purchase, each bottle of wine was subdivided in seven or eight 100-ml vials that were sealed under argon to provide a protective atmosphere until analysis.

**Table 1:** PDO wines included in the study.

PDO Name	Red	White
*Croatian PDO wines*		
Hrvatska Istra	3	9
Slavonija		9
Srednja i Južna Dalmacija	9	
Plešivica	3	4
Dingač	1	
Moslavina	1	
Ponikve	1	
Sjeverna Dalmacija	1	
Zapadna Kontinentalna Hrvatska		1

*Italian PDO wines*		
Chianti	11	
Chianti Classico	6	
Morellino di Scansano	2	
Bolgheri	1	

*Spanish PDO wines*		
Rioja	12	
Ribera del Duero	7	
Bierzo	6	5
Valdeorras		5
Toro	3	
La Mancha	1	
Catalunya	1	

### 2.2 Sample preparation

EDXRF is characterised by limits of quantification (LOQ), around 1 mg kg^−1^ for most elements, and even between 100 and 1000 mg kg^−1^ for Na, Mg, Al, S, Cl and P. Those LOQs are not low enough to carry out direct elemental analyses in wine and a pre-concentration step is needed. For that purpose, 80 mL of wine were evaporated for 5 h under a 0.3 mL/min/sample nitrogen flow, at 60 °C, in a Multivap nitrogen evaporator (Organomation Associates INC (266 River Road West, Berlin, Massachussetts, USA). The pre-concentration was run in triplicate and the viscous residues obtained were weighed and combined to obtained the approximately 10 g of residue needed for the measurement. The resulting paste was transferred to the EDXRF sample holders and used for further analyses.

### 2.3 Determination of the elemental profile of wine by ED-XRF

EDXRF was the technique used for the elemental characterisation of wine because it eliminates the need to treat the sample with hazardous reagents, simplifying the analytical process, and because it allows the straightforward determination of Cl and Br, elements that could be important for classification purposes. Pre-concentration by evaporation would address the issue of the relatively high LOQs that characterise that technique.

EDXRF analyses were carried out using an Epsilon 5 (PANalytical, Almelo, The Netherlands) spectrometer and a method previously optimised and validated for the analysis of organic and inorganic matrices. Calibration curves were constructed using 45 certified reference materials (CRMs) and reference materials (RFs), 23 with an organic matrix and 22 with an inorganic one. The accuracy of the method was evaluated with 25 CRMs and RMs, 13 with organic matrix and 12 with an inorganic one. Information about limit of quantification (LOQ), working range, accuracy, and expanded measurement uncertainties for the different elements, as well as the full list of CRMs and RMs used, can be found elsewhere (Fiamegos & de la Calle Guntiñas, 2018).

### 2.4 Statistical and multivariate analyses

The Student's *t*-test (95 % confidence interval), run to identify the significantly different elemental mass fractions in the different PDOs compared, were carried out with the software Statistica (TIBCO, Version 13.5.0.17).

The software SIMCA Version 15.0.2, Umetrics (Sartorius Stedim Biotech AS, Malmö, Sweden), was used to carry out multivariate analysis of the data (Eriksson et al., 2013).

Partial Least Square Discriminant Analysis (PLS-DA), a supervised technique that maximises the differences between several groups, and hierarchical cluster analyses were used to evaluate if the analysed wines form separated clusters corresponding to the different PDOs. PLS-DA and Soft Independent Modelling by Class Analogy (SIMCA), a supervised technique that maximises the similarities within a group, were used for authentication purposes. The Mahalanobis distance (DModX PS+), distance between a point and the centroid of the distribution, was used to evaluate the ratios false negatives (FN)/true positives (TP) and false positives (FP)/true negatives (TN), in each of the studied models.

The sensitivity, specificity and accuracy of the models was calculated using the following formulas (Barbosa et al., 2016):

$$\text{Sensitivity}=\mathrm{TP}/\left(\mathrm{TP}+\mathrm{FN}\right)$$

$$\text{Specificity}=\mathrm{TN}/\left(\mathrm{TN}+\mathrm{FP}\right)$$

$$\text{Accuracy}=\left(\mathrm{TP}+\mathrm{TN}\right)/\left(\mathrm{TP}+\mathrm{TN}+\mathrm{FP}+\mathrm{FN}\right)$$

where:TP: True positive, TN: True negative, FP: False positive, FN: False negative.

The reduced number of samples available per PDO did not allow the use of part of the samples to train the model, and the rest to test the model performance. For that reason, the sensitivity of the models was calculated by cross-validation leaving one sample out. The specificity was calculated predicting if all the wine samples that did not belong to a certain PDO, were accepted or rejected by the model corresponding to that PDO. It was observed that the performance of the SIMCA authentications improved when the PCA models were built up using exclusively the elements with concentrations above their respective LOQ, in all or in some samples. It was also observed that the performance of the PLS-DA authentications improved when all elements analysed were used to build up the models, probably because in some cases the content of the element was below the LOQ but above the Limit of Detection (LOD) and differences were detected among different categories. It needs to be considered that PLS-DA magnifies differences between groups.

## 3 Results and discussion

### 3.1 Major and trace elements composition of the wines analysed in the study

The concentrations of most elements in wine were too low to be quantified by EDXRF without pre-concentration step. For that reason, samples were evaporated at 60 °C under a nitrogen flow for 5 to 6 h. Only the Mg, P, Cl, S, K, Ca, Cr, Fe, Ni, Rb and Sr concentrations in the product obtained after evaporation were higher than the respective quantification limits (LOQs) of the method, published elsewhere (Fiamegos & de la Calle Guntiñas, 2018), for all or some of the samples. The concentrations of Mg, P, Cl, S, K, Cr, Fe, Ni, Rb and Sr, were significantly different between white and red wines; only the Ni and Rb contents were higher in white than in red wines.

Supplementary 1 summarises information about the wines analysed, among others, PDO, region, country, grape variety, alcohol content, type of barrel used for production, production year, etc. Supplementary 2 summarises the concentrations (raw data) obtained after pre-concentration by evaporation, measurement uncertainties associated to the results, and the LOQs of the applied method. Supplementary 2 only shows the elements that are present at concentrations higher than the respective LOQ in all or some samples.

Table 2 a and b summarises the outcome of the *t*-tests carried out to evaluate which elements are present at significantly different concentrations in the different white and red PDO wines, respectively, when compared in pairs. Most wines within a certain PDO were obtained with grapes from the same variety but there are some exceptions to that rule. Some wines were obtained with grapes of a different variety or with mixtures of several varieties, Supplementary 1. The different varieties within a PDO were pooled together to carry out the t-tests. Only elements with concentrations above the respective LOQs are reported in Table 2.

**Table 2:** Significantly different elements with concentrations above the respective LOQs of the XRF method between: a) PDOs, b) Countries, c) Regions within a country.

A)					
White wine					
PDO	Bierzo	Valdeorras	Slavonija	Hrvatska Istra	Plešivica
n	5	5	8	9	4
Bierzo		Mg, Ni, Ba	Cl, Mn, Ni, Ba	P, Ca, Ba	Cl, S, Mn, Br, Ba
Valdeorras			Ni, Cu, Br	–	Cl, S, Ni, Zn, Br
Slavonija				Cl, Ni	Cl, S, Ca, Mn, Ba
Hrvatska Istra					Cl, S, Mn, Zn, Ba
Plešivica					


Red wine							
PDO	Chianti	Chianti Classico	Italian wines from Toscana not PDO	Bierzo	Rioja	Ribera del Duero	Srednja i Južna Dalmacija
n	12	5	6	6	12	7	9
Chianti		Fe, Ni	K	Mg, P, K, Cr, Mn, Fe, Zn, Rb, Sr, Ba	Mg, P, K, Mn, Fe, Ni, Zn, Br, Rb, Sr, Ba	S, K, Ca, Cr, Mn, Fe, Ni, Zn, Br, Rb	Mg, P, K, Ca, Mn, Fe, Ni, Zn, Br, Rb, Sr, Ba
Chianti Classico			Ni	Mg, P, K, Cr, Mn, Zn, Rb, Sr, Ba	Mg, K, Zn, Rb, Sr, Ba	S, K, Cr, Zn, Rb	Mg, P, Mn, Sr, Ba
Italian from Toscana not PDO				Mg, P, K, Cr, Mn, Zn, Rb, Sr, Ba	Mg, P, K, Mn, Ni, Zn, Rb, Sr	S, K, Cu, Mn, Fe, Ni, Zn, Br, Rb	Mg, P, Mn, Fe, Ni, Zn, Br, Rb, Sr, Ba
Bierzo					Mg, Cr, Mn, Ni, Zn, Rb, Sr, Ba	Mg, S, Mn, Ni, Ba	K, Cr, Mn, Ni, Br, Sr, Ba
Rioja						Fe, Cu, Zn, Rb, Sr	K, Mn, Fe, Br, Rb, Sr, Ba
Ribera del Duero							S, K, Rb, Sr, Ba
Srednja i Južna Dalmacija							


b)			
Red wine			
Country	Croatia	Italy	Spain
n	19	26	32
Croatia		Mg, P, K, Cr, Mn, Fe, Ni, Zn, Br, Sr, Ba	Cl, K, Mn, Rb, Sr, Ba
Italy			Mg, P, K, Cr, Fe, Ni, Zn, Rb, Sr
Spain			


c)		
Country	La Rioja	Castilla y León
n	12	16
La Rioja		Mn, Zn, Rb, Sr, Ba
Castilla y León		

**Fig. 1:**
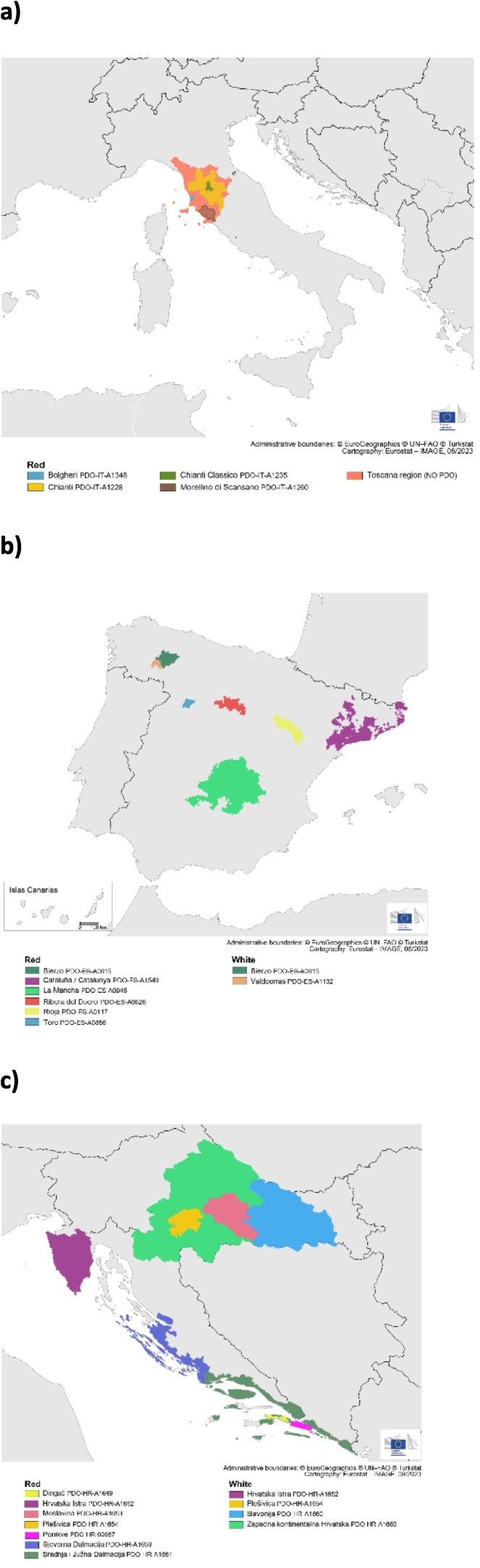
Maps of the PDOs covered in the study: a) Italian, b) Spanish, c) Croatian.

Only in one combination of white wines (Valdeorras-Hrvatska Istra), no elements were present at significantly different concentrations. For the remaining combinations, the lower number of significantly different concentrations was found for the Italian wines (Chianti, Chianti Classico, and red wines from Toscana without PDO claims), and for the two Spanish PDO white wines (Bierzo and Valdeorras). These findings were expected because those groups were included to challenge the study, having regions close to each other where no major differences are expected, neither in the composition of the soils where the grapes are grown, nor in the wine. The area where the Chianti Classico is produced, is situated in the middle of the Chianti area (Fig. 1.a), and all the Italian wines without PDO claims in this study, were according to their labels produced in Toscana. In the case of the Spanish white wines, the two PDO regions are next to each other (Fig. 1.b), but have different climatic conditions; Valdeorras has Mediterranean-Atlantic weather (*e-Ambrosia*, 2024), while Bierzo has a microclimate between Atlantic and continental (Communication, 2020). Fig. 1.a-c shows the geographical zones covered by the PDOs included in the study.

**Fig. 2:**
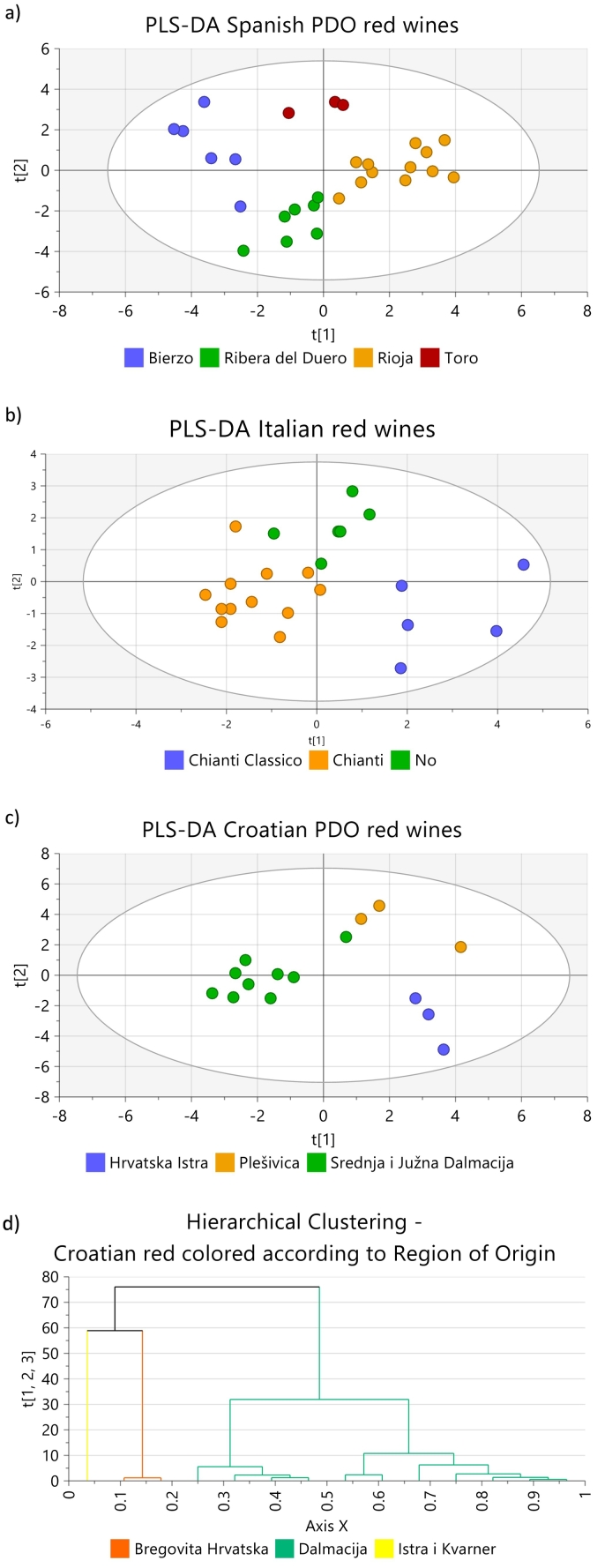

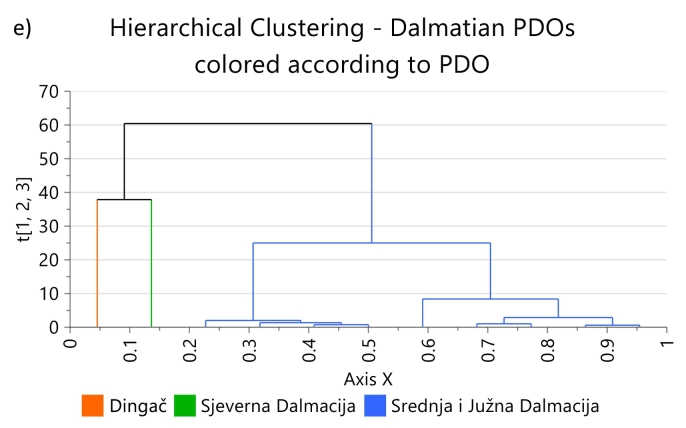
PLS-DA score plots of: a) Spanish PDO red wines, b) Italian red wines, and c) Croatian red wines, represented in the study by three or more samples, d) Hierarchical clustering for Croatian PDO red wines based on region of origin, and e) Hierarchical clustering for Croatian Dalmacian PDO red wines. Ellipse in PLS-DA models: Hotelling's T2 (95 %), Hierarchical clustering calculated with Ward and sorted by size. (For interpretation of the references to colour in this figure legend, the reader is referred to the web version of this article.)

**Fig. 3:**
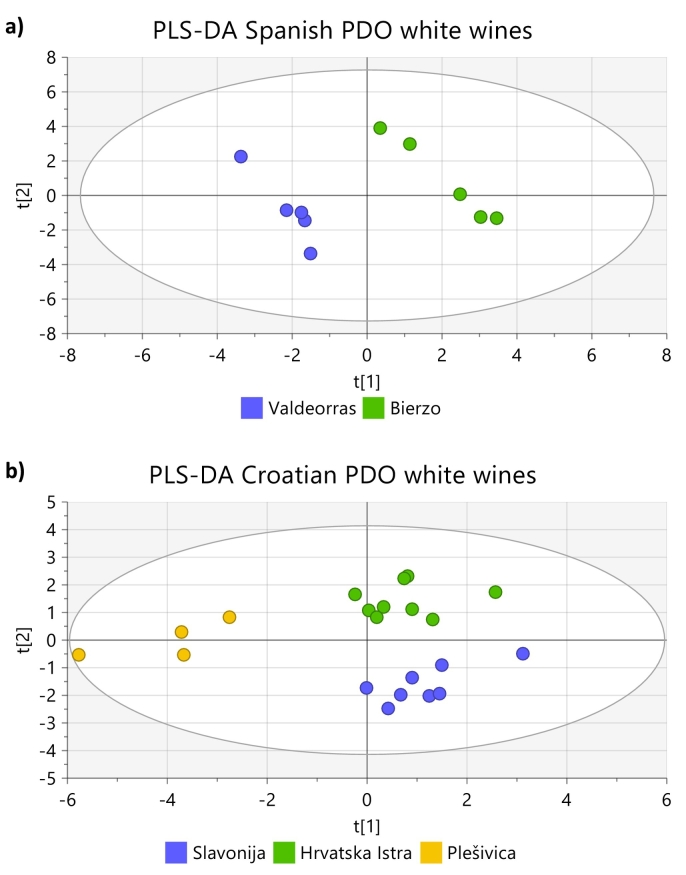
PLS-DA score plots of: a) Spanish PDO white wines, and b) Croatian white wines, represented in the study by three or more samples. Ellipse: Hotelling's T2 (95 %).

As preliminary study, to evaluate if the elemental profiles of wines even coming from close geographical regions, are different enough to be used for authentication purposes, PLS-DA score plots according to PDO, including wines without PDO labels (marked as “No” in the figure), were made for all PDOs for which at least three samples were available. PLS-DA was chosen because it is a multivariate supervised technique that maximises the differences between groups. The PLS-DA score plots, Figs. 2 a-c (red wines) and Figs. 3 a-b (white wines), show that wines from the same PDO cluster together, and separated from the clusters of the other PDOs, regardless of the year of production, grape variety, and production technique. This is true even if the regions covered by the PDO are next to each other such Chianti and Chianti Classico, and Bierzo and Valdeorras, respectively. The only exception to that rule was a Croatian red wine from the Srednja I Južna Dalmacija PDO, which was projected together with the Plešivica wines and not with those of its own PDO, Fig. 2 c. No explanation was found for this behaviour because that wine was produced with grapes of the same variety (Plavac mali) as the other wines from that PDO, and does not have any peculiarity according to the information provided on the label. With few exceptions, the concentrations of most elements in that wine are lower than in the others of the same PDO.

Also wines from PDOs for which few elements have significantly different concentrations, Italian wines from Toscana (Fig. 2 b) and Spanish white wines from Bierzo and Valdeorras (Fig. 3 a), are projected in separated clusters.

Those results were expected because authentication of the geographical origin of wine, is widely documented in the literature (Astray et al., 2021; da Costa et al., 2020; Duley et al., 2021; Ghezzi et al., 2018; Orellana et al., 2019; Rapa et al., 2023; Rodrigues et al., 2020; Wu et al., 2019; Wu et al., 2021; Yamashita et al., 2019).

Biplots of the different PLS-DA models are provided in Supplementary 3 to show the elements that have a strong impact in the formation of the different clusters and make wine classification possible.

### 3.2 Classification studies by soft independent modelling by class analogy

Once demonstrated that the elemental profiles of wines from even close PDOs are different, classification studies for the different PDOs, were carried out. Considering the very large number of different wines commercialised in the world, and which theoretically could be sold with false PDO claims, an attempt was made to use Soft Independent Modelling by Class Analogy for classification purposes. Soft Independent Modelling by Class Analogy would help to decide if a wine sample belongs or not to a certain category, rather than forcing the classification of all samples in one of the categories (Rácz et al., 2018), as PLS-DA does.

Table 3.a shows that the performance of the SIMCA models is not always optimal, with accuracies as poor as 50 % for some PDO's. The specificity of the red wine models varied from 68 % (Chianti Classico) to 98 % (Rioja), and that of the white wines from 44 % (Hrvatska Istra) to 79 % (Bierzo). Thus, 23 out of the 71 non-Chianti Classico red wines, would not have been flagged by the Chianti Classico model as outliers to that model and would be false positives (FP). This can be easily explained because the study includes 26 other wines from Toscana. In addition, 25 non-Hrvatska Istra white wines, would not have been detected as outliers to that model. Few or no elements are present in significantly different concentrations in wines from Hrvatska Istra and other white wines, including those from Spain, what could explain the high number of FP for this PDO.

**Table 3:**
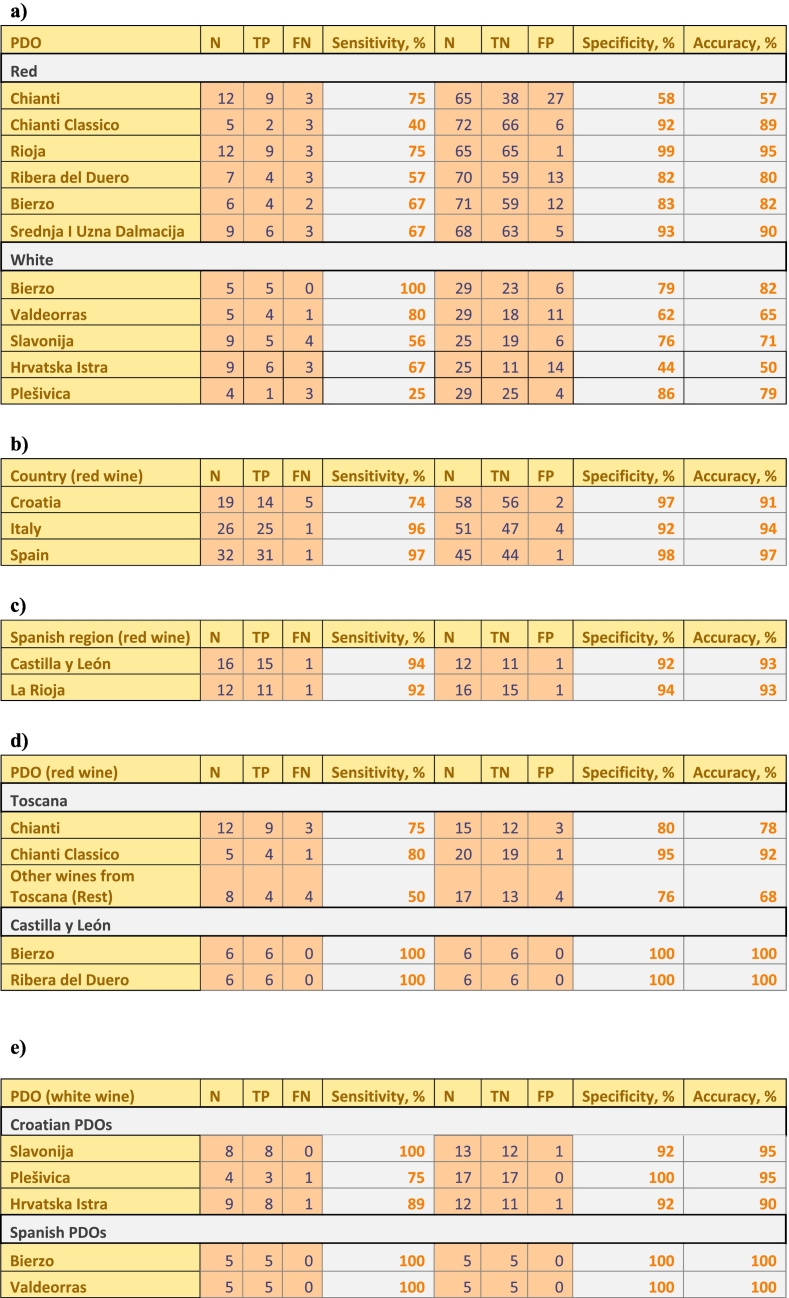
Sensitivity, specificity and accuracy of: a) SIMCA classifications by PDO, b) PLS-DA classifications of red wines by country of origin, c) PLS-DA classifications of red Spanish wines by region of origin, d) PLS-DA classification of red wines by PDO within regions, e) PLS-DA classification of white wines by PDO within countries. (n: Number of samples, TP: True positives, FN: False negatives, TN: True negatives, FP: False positives).

The sensitivity of the red wine models varies from 57 % (Ribera del Duero) to 75 % (Rioja), and for the white wines from 56 % (Slavonija) to 100 % (Bierzo). Hence, 3 out of the 7 Ribera del Duero wines, and 4 out of the 9 Slavonija wines, would have been flagged as outliers by the models of their respective PDO, and would be false negatives (FN).

SIMCA is primarily a class-modelling method and not a discriminant tool, and for this reason its performance for classification purposes is worse than that of other tools used in chemometrics developed for classification purposes (Rácz et al., 2018). In this particular study, the small number of available samples (5 to 12 samples per group), contributes most likely to the poor performance obtained for some of the models. Frequently, hundreds of samples are used for authentication purposes (Hu et al., 2019; Wu et al., 2019). However, it is not realistic that a control laboratory will have access to hundreds of wines with traceability records from each of the more than thousand registered PDOs. Therefore, a different approach should be tested.

### 3.3 Classification studies by partial Least Square discriminant analyses

PLS-DA is known to have a better classification performance than SIMCA, but it has the drawback that it forces observations to be classified in one of the categories included in the model. Unfortunately, PLS-DA works correctly only when applied to a small number of categories, if possible two. This excludes the possibility of using a PLS-DA with more than thousand categories (one for every registered PDO). Ideally, it would be possible to authenticate each wine with a two-category PLS-DA, one category being the PDO of the particular wine to be authenticated, for instance “Rioja”, and the other one all the other wines in the world outside the mentioned PDO, “non-Rioja”. An attempt was made to use this approach but, as expected, the “non-PDO” category was characterised by a very large standard deviation, resulting in a poor performance of the classification model. To address this problem a consecutive classification approach was applied. Wine samples were classified at different levels, first at country level, then at the level of regions within the country, and finally to the PDO within the region. As indicated above, authentication studies were carried out leaving one sample out and classifying it with the models constructed with the remaining samples. For the first level classification (country), a model was created with three categories, Croatian, Italian and Spanish wines, respectively. All the wines included in the study, also those without PDO claims, were used to define the corresponding category. When a wine was classified as belonging to a category (country) different than the one indicated on the label, another PLS-DA model was created only for two categories, the one indicated on the label and the one coming out of the first PLS-DA classification. The second PLS-DA allows the use of PLS-DA at its best, forcing the classification in one out of two categories. For the second PLS-DA, only the elements significantly different in the two categories included in the model, were used as variables, Table 2b. The wines that according to the PLS-DA classification belonged to a category different than the one indicated on the label, FN, were eliminated for further classifications at region and PDO level. Models were constructed with commercially available samples and therefore, it is not possible to exclude the possibility that some wines included in the study were issue of fraud. When a sample is classified as not belonging to the category indicated on the label, it is not possible to know if this is due to a poor performance of the model or if the sample is fraudulent. If the latter applies, the further use of that sample for classification purposes would bias the classification at region and PDO level.

Regarding red wines, one Italian and one Spanish wine were classified as Croatian; one Croatian wine was classified as Spanish and four as Italian. All of them had PDO claims. As indicated in the paragraph above, the misclassifications could be due to a poor performance of the model and be FNs, or to the fact that those wines were not made with grapes cultivated in the country indicated on the label. The sensitivity, specificity and accuracy of the classification of wines per country of origin using the approach described above, is given in Table 3 b.

Unfortunately, after removing the five Croatian red wines that were incorrectly classified, the only Croatian region with more than 4 samples in the study was Dalmacija, and within that region the only PDO represented by more than 4 samples was Srednja i Južna Dalmacija. For this reason, no further authentication studies could be carried out on Croatian red wines. The hierarchical clustering of Croatian red wines by region, and by PDOs within the Dalmacja region, Figs. 2 d and e respectively, show a good separation in clusters for the different regions and PDOs. Nevertheless, considering the reduced number of observations (samples), those figures can only be considered as indicative, and a proper validation leaving one sample out should be carried out, including a high number of samples representing the wider variability that can be expected for the different categories.

For the second level of classification according to regions, a PLS-DA model was created for regions within the country to which the wine belonged. For instance, all the Spanish PDOs for which more than four samples were included in the study, were coming from two regions, La Rioja and Castilla y León, respectively. Accordingly, a PLS-DA model was created for those two regions, excluding from the model all Spanish wines coming from other regions. Also PDO/PGIs from Castilla y León represented in the study by less than 4 samples (Toro and Vino de la Tierra Castilla y León) were taken to build up the Castilla y León category. Again in this case the two-category PLS-DA model, was constructed using only as variables the elements with significantly different concentrations in the two categories, Table 2 c. The samples classified in a category (region) different from the one indicated in the label, were eliminated from further classifications at PDO level. One wine from La Rioja was misclassified as belonging to Castilla y León and one wine from Castilla y León was misclassified as coming from La Rioja, Table 3 c. All red wines from Italy, came from Toscana and for that reason the second classification level was not needed.

For the third level, PLS-DA models were created for the different PDOs within the different regions for which more than four samples were available, Table 2 a and Table 3 d. For instance, in the case of Castilla y León, two PDOs were covered in the study with enough red wines samples, Bierzo and Ribera del Duero. All wines were properly classified in their own category (PDO). The PLS-DA model classified one Bierzo wine as belonging to Ribera del Duero. However, this is a special case in which one single element, manganese, allows to differentiate wines from Bierzo from wines from Ribera del Duero. It is known that the Bierzo area is rich in Mn (Ghidotti et al., 2021; Gómez-Miguel et al., 2014). All wines from the Bierzo PDO have a Mn mass fraction higher than 1 mg kg^−1^, while all Ribera del Duero had Mn mass fractions below 1 mg kg^−1^. Based on the Mn content it was decided to consider the mentioned Bierzo sample as a TP for the Bierzo category. The Mn content in Bierzo wines is not significantly different from other wines included in the study coming from zones close to El Bierzo, such as wines with Toro PDO and with Vino de la Tierra Castilla y León PGI. Two differentiate wines from such close regions the number of wines from every PDO/PGI zone needs to be increased and the wines need be chosen to cover the widest variability expected within the PDO/PGI.

For the wines from Toscana three categories were included in the third level classification according to PDO: Chianti, Chianti Classico, and wines produced in Toscana without PDO claims, or belonging to PDOs for which less than four samples were included in the study (Morellino di Scansano, and Bolgheri), referred to as “Rest”. The wines wrongly classified in this three-category PLS-DA model, were classified using a two-category PLS-DA model constructed for the PDO to which the wine belongs according to the model and for the PDO indicated in the three category initial PLS-DA model. Only significantly different elements were used in the construction of the two-category PLS-DA model. One sample labelled as Chianti Classico was classified as belonging to the Rest category and one wine from the Rest category was wrongly classified as Chianti Classico. The differentiation between Chianti Classico and Chianti using the three-category PLS-DA was 100 % accurate. The differentiation between the wines with Chianti PDO and the wines in the Rest category was not fully satisfactory; three wines with the Chianti label were classified as Rest and four wines belonging to the Rest category, were classified as Chianti, Table 3 d. Some PDOs are divided in different categories and sub-zones, each one with different general analytical characteristics. This is the case of the Chianti PDO with nine subdivisions: Chianti, Chianti Superiore and seven sub-zones. Among the 12 Chianti wines included in this study, 7 were labelled as Chianti, 2 as Chianti Superiore, 1 as Chianti Mostespertoli, 1 as Rufina, and 1 as Colle Senesi. In a PCA plot of the Chianti wines (not shown), the Chianti Montespertoli wine was projected quite separated from the other Chianti wines, increasing the standard deviation of the model what could be responsible for the high rate of FP in the Chianti category, using both, the SIMCA and the PLS-DA approach. Ideally, an independent model should be made for each of the sub-zones/categories. The possibility of using the elemental profiles of wine to authenticate PDO claims, was tested at its most in this study with the Italian wines. All Italian red wines came from the same region, covering PDO areas quite close to each other, even with one PDO area, Chianti Classico, being situated in the centre of another one, Chianti, Fig. 1 a, and including wines produced in Toscana but without PDO claims. Some of the brands included in the Rest category produce also wines with Chianti PDO label; thus the possibility of mixtures of grapes from the PDO zone and from other areas in Toscana to produce wines without PDO label, cannot be excluded. For cases like the classification of the Italian red wines in this study, the use of other techniques such as NMR, GC–MS, HPLC-MS, etc., could be extremely useful for authentication purposes, extending the amount and type of chemical compounds to be used as variables. PDOs covering large zones (large standard deviation in the models built-up with elemental profiles), would benefit particularly of that approach. It also needs to be considered, that some wine producers located in a certain PDO area might chose not to use the PDO label on their products to avoid administrative burden, putting on the market wines that fulfil all the quality criteria of the PDO wines from the same area, also in terms of chemical composition. For this reason, traceability controls based on the evaluation of the relevant documentation will always be necessary, and cannot be fully replaced by laboratory analyses.

Regarding the classification of white wines, only Croatian and Spanish wines were included in this study. It did not make sense to classify the wines according to country of origin because the two Spanish PDOs only cover two consecutive small zones not even representing a full province. Neither the classification at regional level within Spanish and Croatian wines made sense because each one of the regions included in the study, were covered by only one PDO. For this reason, the only classification that could be done was the third level classification, comparing Spanish PDOs between them, and Croatian PDOs among them, Table 3 e. One sample with Slavonija PDO was an outlier being clearly projected outside the 95 % confidence interval in the model; for this reason, that sample was excluded from the classification exercise. All the other samples with Slavonija PDO labels were correctly classified. One sample with Plešivica PDO label was classified as Hrvatska Istra, and one Hrvatska Istra wine as Slavonija. All Spanish wines were classified correctly.

As expected, both for the red and the white wines, the performance of the PLS-DA classification was better than that of SIMCA. On average, the sensitivity improved from 65 % to 91 %, and the specificity from 80 % to 89 %.

### 3.4 Challenges of a successful control system

A drawback of the approach reported here is that one or several of the wines analysed could be counterfeited because all of them were purchased at supermarkets and retailers, and traceability records were not available. Those samples would bias the results obtained, increasing the standard deviation of the model constructed for the PDO indicated on their label.

A way to address the limited amount of samples and the lack of traceability, would be to use centralised databases containing records for a large number of representative samples, not only for a given PDO but also for non-PDO wines produced in other countries and even continents, and that could be accessed by control authorities. However, who should own and populate such databases? The PDO consortia? Private service providers? The EU as owner of the eAmbrosia database and the PDO concept? The countries in which the different PDOs are located?

In case the classification performance of SIMCA would increase when models are constructed with a large number of samples, what would need to be demonstrated, the first option would be probably the most feasible. PDO consortia would be responsible for the construction of the database and commit to support control laboratories in case of suspicion of counterfeit, namely analysing the suspicious sample and stating if it belongs or not to the PDO category, in other words, if it is genuine or not. An example of this approach in an area different from wine, is the case of the Formaggio Parmigiano-Reggiano cheese (Camin et al., 2012); the consortium of that PDO published a paper on the H, C, N and S stable isotopes and mineral profiles of different hard cheeses to warranty the authenticity of Parmigiano Reggiano. Consortia have access to a large number of samples with traceability records. After an initial phase, each consortium would have to analyse 30 to 50 samples each year, which is the amount of samples recommended to validate a PCA classification method (Gemperline, 2006). Many commercial laboratories are accredited for the analyses of major and trace elements and it would not be difficult for the consortia to outsource the analyses. The drawback of this approach is that controls would be needed to ensure the transparency of the system, guaranteeing that only representative samples from the PDO are used to populate the database and that collusion between the consortia and private laboratories does not take place. The intellectual property rights on PDO wines are owned by the respective consortia, and this approach would avoid disputes on who can access the data.

The last two options are probably the most trustworthy options. They are also the only feasible option if the consecutive PLS-DA cascade is chosen as classification tool. A central and common database would be constructed by a network of authorised laboratories in the Member States, laboratories that would have to take part regularly in proficiency tests to demonstrate that their results are comparable. The network could be coordinated by an international organisation, for instance by the European Commission, that hosts already the European Reference Centre for Control in the Wine Sector. The coordinator should also control the access to the database. The sampling could even take place at the vineyards, assuming that the processing of the wine does not have much impact on the elemental profile of the final product and that micro vinified wines processed by control laboratories could be used to populate the databases; nevertheless, this would have to be proven with proper studies. Sample collection could also take place at the producer as part of the control plans of each country, or to avoid problems about the ownership of the data, wines could be bought to the producers. In case of doubt about the authenticity of a sample, analyses with more sophisticated techniques such as NMR, GC–MS, HPLC-MS, etc., could be performed before deciding if the sample should be kept in the database or not. However, countries such as Italy or France with 410 and 366 currently registered wine PDOs, respectively, would have to do hundreds of thousands of analyses, if not every year, at least in an initial phase, and several hundred each year to cover for seasonal variations. A way to reduce the amount of analyses would be to contribute to the database for a particular PDO only when there is the suspicion that wines of that PDO are being counterfeited. Even then, a minimum amount of samples per country, per region within the different countries, and per wine producing zones within a region, would have to be analysed in an initial phase to construct the consecutive PLS-DA models. Also, wines from other continents would have to be analysed and the results registered in the database, to be able to identify replacement of PDO wines by cheaper ones produced in other parts of the world, not only in Europe.

In any case, to implement the Official Controls Regulation (*Regulation (EU*) 2017/625 of the European Parliament and of the Council of 15 March 2017 on official controls and other official activities performed to ensure the application of food and feed law, rules on animal health and welfare, plant health and plant protection products, 2017), a system needs to be built up and covered by legislation, with clear definitions of who are the actors and their rights and obligations.

## 4 Conclusions

The geographical indication concept is an important pillar of the socio-economic sustainability of rural areas. The list of wines holding PDO or PGI labels, registered in e-Ambrosia, keeps growing. The willingness of consumers to pay more for products of recognised quality is probably one of the main explanations for that trend. To avoid fraudsters to make economic profit out of this trend, effective control tools need to be put in place.

Laboratory controls and controls of document traceability records are the two pillars on which the control system should be constructed. In this work, the feasibility of using elemental profiles to authenticate PDO claims in the wine sector, has been demonstrated. The technique used, ED-XRF, is fast and environmentally friendly because it does not imply the use of hazardous chemicals. Nevertheless, even if the analytical tools are there, given the size of the wines business, international collaboration is needed to build up robust databases with a broad representation of commercially available wines.

## CRediT authorship contribution statement

**Michele Ghidotti:** Writing – review & editing, Validation, Investigation, Formal analysis. **Sergej Papoci:** Formal analysis. **Arantza Respaldiza:** Writing – review & editing, Conceptualization. **Håkan Emteborg:** Writing – review & editing, Formal analysis. **Franz Ulberth:** Writing – review & editing, Conceptualization. **María Beatriz de la Calle Guntiñas:** Writing – original draft, Validation, Supervision, Methodology, Investigation, Data curation, Conceptualization.

## Declaration of competing interest

The authors declare that they have no known competing financial interests or personal relationships that could have appeared to influence the work reported in this paper.

## Data Availability

Data provided in Supplemetary 2

## Appendix A Supplementary data

Supplementary material 1 Image 1

Supplementary material 2 Image 2

Supplementary material 3: Biplots of the different PLS-DA models. Image 3

## References

[bb0005] Aceto M., Gulino F., Calà E., Robotti E., Petrozziello M., Tsolakis C., Cassino C. (2020). Authentication and traceability study on Barbera d’Asti and Nizza DOCG wines: The role of trace and ultra-trace elements. Beverages.

[bb0010] Astray G., Martinez-Castillo C., Mejuto J.C., Simal-Gandara J. (2021). Metal and metalloid profile as a fingerprint for traceability of wines under any Galician protected designation of origin. Journal of Food Composition and Analysis.

[bb0015] Barbosa R., Silva de Paula E., Paulelli A.C., Moore A.F., Oliveira Souza J.M., Lemos Batista B., Barbosa J.R. (2016). Recognition of organic rice samples based on trace elements and support vector machines. Journal of Food Composition and Analysis.

[bb0020] Camin F., Wehrens R., Bertoldi D., Bontempo L., Ziller L., Perini M., Nicolini G., Nocetti M., Larcher R. (2012). H, C, N and S stable isotopes and mineral profiles to objectively guarantee the authenticity of grated hard cheeses. Analytica Chimica Acta.

[bb0025] Catarino S., Madeira M., Monteiro F., Caldeira I., de Sousa R.B., António Curvelo-Garcia A. (2018). Mineral composition through soil-wine system of Portuguese vineyards and its potential for wine traceability. Beverages.

[bb0030] Communication (2020).

[bb0035] da Costa N.L., Bianchi Ximenez J.P., Lisboa Rodrigues J., Barbosa F., Barbosa R. (2020). Characterization of cabernet sauvignon wines from California: Determination of origin based on ICP-MS analysis and machine learning techniques. European Food Research and Technology.

[bb0040] Duley G., Dujourdy L., Klein S., Werwein A., Spartz C., Gougeon R.D., Taylor D.K. (2021). Regionality in Australian pinot noir wines: A study on the use of NMR and ICP-MS on commercial wines. Food Chemistry.

[bb0045] Dumitrascu C., Fiamegos Y., de la Calle Guntiñas M.B. (2021). Feasibility study on the use of elemental profiles to authenticate aromatic rice: The case of basmati and Thai rice. Analytical and Bioanalytical Chemistry.

[bb0050] (2024). e-Ambrosia. https://ec.europa.eu/geographical-indications-register/eambrosia-public-api/api/v1/attachments/39186.

[bb0055] Eriksson L., Byrne T., Johansson E., Trygg J., Vikström C. (2013).

[bb0060] EURACHEM, Bettencourt da Silva R., Ellison S.L.R. (2021). / CITAC guide. Assessment of performance and uncertainty in qualitative chemical analysis.

[bb0065] Farm to Fork Strategy (2024). For a fair, healthy and environmentally-friendly food system. https://food.ec.europa.eu/horizontal-topics/farm-fork-strategy_en.

[bb0070] Fiamegos Y., de la Calle Guntiñas M.B. (2018). Validation strategy for an ed-xrf method to determine trace elements in a wide range of organic and inorganic matrices based on fulfilment of performance criteria. Spectrochimica Acta Part B.

[bb0075] Fiamegos Y., Dumitrascu C., Ghidotti M., de la Calle Guntiñas M.B. (2020). Use of energy-dispersive X-ray fluorescence combined with chemometric modelling to classify honey according to botanical variety and geographical origin. Analytical and Bioanalytical Chemistry.

[bb0080] Fiamegos Y., Dumitrascu C., Papoci S., de la Calle M.B. (2021). Authentication of PDO paprika powder (Pimentón de la Vera) by multivariate analysis of the elemental fingerprint determined by ED-XRF. A feasibility study. Food Control.

[bb0085] Gemperline P. (2006).

[bb0090] Ghezzi L., Arienzo I., Buccianti A., Demarchi G., Petrini R. (2018). Highly radiogenic Sr-isotopic signature and trace element content of grape musts from northern Piedmont vineyards (Italy). European Food Research and Technology.

[bb0095] Ghidotti M., Fiamegos Y., Dumitrascu C., de la Calle M.B. (2021). Use of elemental profiles to verify geographical origin and botanical variety of Spanish honeys with a protected denomination of origin. Food Chemistry.

[bb0100] Gómez-Miguel V.D., Ruiz V.S., V. (2014).

[bb0105] Grindlay G., Mora J., Grasa L., de Loos-Vollebregt M.T.C. (2011). Atomic spectrometry methods for wine analysis: A critical evaluation and discussion of recent applications. Analytica Chimica Acta.

[bb0110] Hu X.Z., Liu S.Q., Li X.H., Wang C.X., Ni X.L., Liu X., Xu C.H. (2019). Geographical origin traceability of cabernet sauvignon wines based on infrared fingerprint technology combined with chemometrics. Scientific Reports.

[bb0115] Orellana S., Johansen A.M., Gazis C. (2019). Geographic classification of U.S. Washington state wines using elemental and water isotope composition. Food Chemistry.

[bb0120] Rácz A., Gere A., Bajusz D., Héberger K. (2018). Is soft independent modelling of class analogies a reasonable choice for supervised pattern recognition?. RSC Advances.

[bb0125] Ranaweera R.K.R., Gilmore A.M., Capone D.L., Bastian S.E.P., S.E.P., Jeffery, D.W. (2021). Authentication of the geographical origin of Australian cabernet sauvignon wines using spectrofluorometric and multi-element analyses with multivariate statistical modelling. Food Chemistry.

[bb0130] Rapa M., Ferrante M., Rodushkin I., Paulukat C., Conti M.E. (2023). Venetian protected designation of origin wines traceability: Multi-elemental, isotopes and chemometric analysis. Food Chemistry.

[bb0135] (2012). Regulation (EU) 1151/2012 of the European Parliament and of the Council of 21 November 2012 on quality schemes for agricultural products and foodstuffs.

[bb0140] (2017). Regulation (EU) 2017/625 of the European Parliament and of the Council of 15 March 2017 on official controls and other official activities performed to ensure the application of food and feed law, rules on animal health and welfare, plant health and plant protection products.

[bb0145] Rodrigues N.P., Rodrigues E., Celso P.G., Kahmann A., Yamashita G.H., Anzanello M.J., Manfroi V., Hertz P.F. (2020). Discrimination of sparkling wines samples according to the country of origin by ICP-OES coupled with multivariate analysis. LWT - Food Science and Technology.

[bb0150] Wehling P., Labudde R.A., Brunelle S.L., Nelson M.T. (2011). Probability of detection (POD) as a statistical model for the validation of qualitative methods. Journal of AOAC International.

[bb0155] Wu H., Lin G., Tian L., Yan Z., Yi B., Bian X., Jin B., Xie L., Zhou H., Rogers K.M. (2021). Origin verification of French red wines using isotope and elemental analyses coupled with chemometrics. Food Chemistry.

[bb0160] Wu H., Tian L., Chen B., Jin B., Tian B., Xie L., Rogers K.M., Lin G. (2019). Verification of imported red wine origin into China using multi isotope and elemental analyses. Food Chemistry.

[bb0165] Yamashita G.H., Anzanello M.J., Soares F., Rocha M.K., Fogliatto F.S., Rodrigues N.P., Rodrigues E., Celso P.G., Manfroi V., Hertz P.F. (2019). Hierarchical classification of sparkling wine samples according to the country of origin based on the most informative chemical elements. Food Control.

