# Supplementary material for: Use of energy dispersive X-ray fluorescence to authenticate European wines with protected designation of origin. Challenges of a successful control system based on modelling
Source: Food Chem. 2025 Feb 15;465:141989. doi: 10.1016/j.foodchem.2024.141989 (PMC11649527; doi:10.1016/j.foodchem.2024.141989)
Supplement: Supplementary file 1 — Supplementary material 1 [file mmc1.docx]

**Supplementary 1:** Information about the wines included in the study as provided on the labels.

| **ID** | **Wine colour** | **PDO/ Not PDO** | **Geographical Indication name** | **Country  of Origin** | **Region  of Origin** | **Grape  variety** | **Production  year** | **Country  of purchase** | **Type  of wine** | **Alcohol  content (%)** | **Barrel** | **Comments** |
| --- | --- | --- | --- | --- | --- | --- | --- | --- | --- | --- | --- | --- |
| 1 | Red | Yes | Rioja | Spain | La Rioja | 60 % Graciano 20 % Garnacha 20 % Tempranillo | 2019 | Belgium |  | 14.5 | n.a. |  |
| 2 | Red | Yes | Rioja | Spain | La Rioja | Tempranillo | 2017 | Belgium | Crianza | 14 | American and French Oak (12) |  |
| 3 | Red | Yes | Chianti  Classico | Italy | Toscana | n.a. | 2019 | Italy |  | 13.5 | n.a. |  |
| 4 | Red | Yes | Morellino di Scansano | Italy | Toscana | n.a. | 2020 | Italy |  | 13 | n.a. |  |
| 5 | Red | Yes | Chianti | Italy | Toscana | n.a. | 2019 | Italy |  | 13 | n.a. |  |
| 6 | Red | Yes | Bierzo | Spain | Castilla  y León | Mencía | 2020 | Belgium |  | 13.5 | n.a. |  |
| 7 | Red | Yes | Chianti | Italy | Toscana | Bacca Rossa | 2019 | Italy |  | 13 | n.a. |  |
| 8 | Red | Yes | Ribera  del Duero | Spain | Castilla  y León | Tempranillo | 2017 | Belgium | Crianza | 14.5 | American and French Oak (12) |  |
| 9 | Red | Yes | Morellino di Scansano | Italy | Toscana | n.a. | 2020 | Italy |  | 13.5 | n.a. |  |
| 10 | Red | Yes | Toro | Spain | Castilla  y León | Tempranillo | 2019 | Belgium |  | 13.5 | n.a. |  |
| 12 | Red | Yes | Bolgheri | Italy | Toscana | n.a. | 2018 | Italy |  | 14 | n.a. |  |
| 13 | Red | Yes | Chianti | Italy | Toscana | Sangiovese Canaiolo | 2018 | Italy |  | 13.5 | n.a. |  |
| 14 | Red | Yes | Rioja | Spain | La Rioja | Tempranillo | 2016 | Belgium | Reserva | 14 | American and French Oak (12) |  |
| 15 | Red | Yes | Rioja | Spain | La Rioja | Tempranillo | 2016 | Belgium | Reserva | 13.5 | Barrel (12) | |
| 16 | Red | No | No | Spain | Castilla- La Mancha | Tempranillo | 2020 | Belgium |  | 12.5 | n.a. |  |
| 17 | Red | Yes | Chianti Superiore | Italy | Toscana | Sangiovese Mixture | 2019 | Italy | Chianti | 13 | n.a. |  |
| 18 | Red | No | No | Italy | Toscana | Ciliegiolo Mixture | 2018 | Italy |  | 13 | n.a. |  |
| 19 | Red | Yes | Rioja | Spain | La Rioja | Tempranillo | 2019 | Belgium |  | 14 | n.a. |  |
| 20 | Red | Yes | Chianti Rufina | Italy | Toscana | n.a. | 2017 | Italy | Riserva | 13.5 | n.a. |  |
| 21 | Red | Yes | Rioja | Spain | La Rioja | Garnacha & Tempranillo | 2019 | Belgium |  | 13 | n.a. |  |
| 22 | Red | Yes | Ribera  del Duero | Spain | Castilla  y León | Tempranillo | 2015 | Belgium | Reserva | 14 | American and French Oak (12) |  |
| 23 | Red | Yes | Toro | Spain | Castilla  y León | Tempranillo | 2019 | Belgium |  | 14.5 | New barrel French Oak |  |
| 24 | Red | Yes | Chianti Superiore | Italy | Toscana | n.a. | 2019 | Italy | Chianti | 13 | n.a. |  |
| 25 | Red | No | No | Italy | Toscana | Merlot Mixture | 2018 | Italy |  | 13 | n.a. |  |
| 26 | Red | Yes | Ribera  del Duero | Spain | Castilla  y León | Tempranillo | 2011 | Belgium | Gran Reserva | 14 | American and French Oak (24) |  |
| 27 | Red | No | No | Italy | Toscana | n.a. | 2018 | Italy |  | 13.5 | n.a. |  |
| 28 | Red | Yes | Chianti | Italy | Toscana | Sangiovese | 2020 | Italy |  | 12 | n.a. |  |
| 29 | Red | Yes | Rioja | Spain | La Rioja | Tempranillo | 2012 | Belgium | Gran Reserva | 13.5 | American and French Oak (24) |  |
| 30 | Red | No | No | Italy | Toscana | n.a. | 2019 | Italy |  | 13 | n.a. |  |
| 31 | Red | Yes | Rioja | Spain | La Rioja | Tempranillo | 2015 | Belgium | Reserva | 13 | Oak (12) |  |
| 32 | Red | Yes | Toro | Spain | Castilla  y León | Tempranillo | 2019 | Belgium |  | 14.5 | Oak (4) |  |
| 33 | Red | Yes | Chianti | Italy | Toscana | Sangiovese Mixture | 2020 | Italy |  | 12.5 | n.a. |  |
| 34 | Red | No | No | Italy | Toscana | Cabernet Mixture | 2018 | Italy |  | 13.5 | n.a. |  |
| 35 | Red | Yes | Ribera  del Duero | Spain | Castilla  y León | Tempranillo | 2017 | Belgium | Crianza | 14 | American oak  (14) |  |
| 36 | Red | Yes | Chianti Colli Senesi | Italy | Toscana | n.a. | 2020 | Italy |  | 13 | n.a. |  |
| 37 | Red | Yes | Catalunya | Spain | Cataluña | Tempranillo | 2018 | Belgium |  | 13.5 | n.a. |  |
| 38 | Red | Yes | Ribera  del Duero | Spain | Castilla  y León | Tempranillo | 2018 | Belgium | Crianza | 14.5 | American and French Oak (12) |  |
| 39 | Red | Yes | Rioja | Spain | La Rioja | Tempranillo | 2009 | Belgium | Gran Reserva | 13.5 | Oak (24) |  |
| 41 | Red | Yes | Rioja | Spain | La Rioja | Graciano | 2018 | Belgium |  | 13.5 | n.a. |  |
| 42 | Red | No | Castilla y León (PGI) | Spain | Castilla  y León | Tempranillo | 2020 | Belgium |  | 13 | n.a. |  |
| 43 | Red | Yes | Chianti  Classico | Italy | Toscana | n.a. | 2018 | Italy | Classico | 13 | n.a. |  |
| 44 | Red | Yes | Chianti  Classico | Italy | Toscana | n.a. | 2018 | Italy | Classico | 13 | n.a. |  |
| 45 | Red | Yes | Rioja | Spain | La Rioja | Tempranillo | 2017 | Belgium | Crianza | 14 | Oak (12) |  |
| 46 | Red | Yes | La Mancha | Spain | Castilla- La Mancha | Tempranillo | 2019 | Belgium |  | 12 | n.a. |  |
| 47 | Red | No | No | Italy | Toscana | Merlot Mixture | 2018 | Italy |  | 13.5 | n.a. |  |
| 48 | Red | Yes | Chianti | Italy | Toscana | n.a. | 2018 | Italy | Riserva | 13.5 | n.a. |  |
| 49 | Red | Yes | Rioja | Spain | La Rioja | Tempranillo | 2017 | Belgium | Crianza | 14.5 | American oak  (12) |  |
| 51 | Red | Yes | Chianti | Italy | Toscana | Sangiovese Mixture | 2017 | Italy | Riserva | 13 | n.a. |  |
| 52 | Red | Yes | Ribera  del Duero | Spain | Castilla  y León | n.a. | 2019 | Spain |  | 14 | n.a. | San Esteban  de Gormaz (Soria) |
| 53 | Red | Yes | Chianti Montespertoli | Italy | Toscana | Sangiovese Merlot Cabernet Sauvignon | 2017 | Italy | Riserva | 14 | n.a. |  |
| 54 | Red | Yes | Hrvatska Istra | Croatia | Istra i Kvarner | Borgonja | 2019 | Croatia |  | 13 | n.a. |  |
| 55 | Red | Yes | Srednja i Južna Dalmacija | Croatia | Dalmacija | Plavac mali | 2019 | Croatia |  | 14 | n.a. |  |
| 56 | Red | Yes | Ribera  del Duero | Spain | Castilla  y León | 88 % tempranillo,  5 % albillo,  7 % garnacha | 2018 | Spain |  | 14 | n.a. | Tubilla del Lago  (Burgos), 900 m |
| 57 | White | Yes | Slavonija | Croatia | Slavonija i hrvatsko Podunavlje | Graševina | 2021 | Croatia |  | 13 | n.a. |  |
| 58 | White | Yes | Valdeorras | Spain | Galicia | Godello | 2019 | Spain |  | 13.5 | n.a. |  |
| 59 | Red | Yes | Bierzo | Spain | Castilla  y León | n.a. | 2016 | Spain |  | 14 | n.a. | Villafranca del Bierzo |
| 60 | White | Yes | Hrvatska Istra | Croatia | Istra i Kvarner | Malvazija | 2021 | Croatia |  | 13.5 | n.a. | Hand picked, hand selected |
| 61 | Red | Yes | Bierzo | Spain | Castilla y León | Mencía | 2020 | Spain |  | 14 | n.a. | Castro Ventosa Valtuille |
| 62 | Red | Yes | Ponikve | Croatia | Dalmacija | Plavac mali | 2018 | Croatia |  | 13.5 | Slavonian oak (24) | BIO |
| 63 | White | Yes | Zapadna kontinentalna Hrvatska | Croatia | Bregovita Hrvatska | Silvanac zeleni | 2020 | Croatia |  | 12 | n.a. |  |
| 64 | White | Yes | Bierzo | Spain | Castilla  y León | Godello | 2019 | Spain | Vino de paraje | 14 | French oak (12) | Cacabelos. Sandy soil. Rich in quartz |
| 65 | White | Yes | Bierzo | Spain | Castilla  y León | Godello | 2020 | Spain |  | 13.5 | n.a. | BIO Valtuille de Arriba |
| 66 | Red | Yes | Hrvatska Istra | Croatia | Istra i Kvarner | Teran | 2018 | Croatia |  | 13 | n.a. |  |
| 67 | Red | Yes | Bierzo | Spain | Castilla  y León | Mencía | 2019 | Spain | Vino de villa | 13.5 | n.a. | San Pedro de  Olleros. (Ancares), 750 m. |
| 68 | White | Yes | Slavonija | Croatia | Slavonija i hrvatsko Podunavlje | Sauvignon Blanc | 2020 | Croatia |  | 13 | The wine was fermented and aged in stainless steel tanks. 9% of the wine was fermented in oak barrels and aged on lees by sur lie method. | The grapes were cold macerated for 18-24 hours. |
| 69 | Red | Yes | Srednja i Južna Dalmacija | Croatia | Dalmacija | Tribidrag (a.k.a. *Zinfandel*, a.k.a. *Primitivo*) + Tempranillo | 2019 | Croatia |  | 13.5 | Barrique | BIO |
| 70 | White | Yes | Valdeorras | Spain | Galicia | Godello | 2020 | Spain | Limited edition | 13.5 | n.a. |  |
| 71 | White | Yes | Plešivica | Croatia | Bregovita Hrvatska | Plavec žuti, Šipelj, Štajerska belina i Kraljevina | 2019 | Croatia |  | 13 | n.a. |  |
| 72 | White | Yes | Valdeorras | Spain | Galicia | Godello | 2019 | Spain |  | 13 | n.a. |  |
| 73 | White | Yes | Hrvatska Istra | Croatia | Istra i Kvarner | Malvazija | 2021 | Croatia |  | 13 | n.a. |  |
| 74 | Red | Yes | Bierzo | Spain | Castilla  y León | Mencía | 2018 | Spain |  | 13.5 | American and French Oak (6) | Very old plants, clay and slate 400-600 m |
| 75 | White | Yes | Slavonija | Croatia | Slavonija i hrvatsko Podunavlje | Graševina | 2019 | Croatia |  | 13 | n.a. | Unfiltered |
| 76 | White | Yes | Bierzo | Spain | Castilla  y León | Godello | 2019 | Spain |  | 13.5 | n.a. |  |
| 77 | Red | Yes | Dingač | Croatia | Dalmacija | Dingač | 2018 | Croatia |  | 16 | n.a. |  |
| 78 | White | Yes | Bierzo | Spain | Castilla  y León | Godello | 2020 | Spain |  | 13.5 | n.a. |  |
| 79 | White | Yes | Valdeorras | Spain | Galicia | Godello | 2021 | Spain |  | 13 | n.a. |  |
| 80 | Red | Yes | Hrvatska Istra | Croatia | Istra i Kvarner | Teran | 2020 | Croatia |  | 13 | n.a. | Hand picked, hand selected |
| 81 | Red | Yes | Moslavina | Croatia | Bregovita Hrvatska | Frankovka | 2017 | Croatia |  | 12.5 | n.a. | Unfiltered |
| 82 | Red | Yes | Bierzo | Spain | Castilla  y León | n.a. | 2019 | Spain |  | 13.5 | n.a. |  |
| 83 | Red | Yes | Srednja i Južna Dalmacija | Croatia | Dalmacija | Plavac mali | 2019 | Croatia |  | 14.5 | n.a. |  |
| 84 | White | Yes | Bierzo | Spain | Castilla  y León | Godello | 2019 | Spain |  | 13.5 | n.a. |  |
| 85 | White | Yes | Hrvatska Istra | Croatia | Istra i Kvarner | Malvazija | 2021 | Croatia |  | 13 | n.a. |  |
| 86 | White | Yes | Valdeorras | Spain | Galicia | Godello | 2019 | Spain |  | 12.5 | n.a. | Mountain wine |
| 87 | Red | Yes | Sjeverna Dalmacija | Croatia | Dalmacija | Babić | 2017 | Croatia |  | 13.5 | n.a. | BIO |
| 88 | White | Yes | Hrvatska Istra | Croatia | Istra i Kvarner | Malvazija | 2021 | Croatia |  | 13 | n.a. |  |
| 89 | Red | Yes | Srednja i Južna Dalmacija | Croatia | Dalmacija | Plavac mali | 2018 | Croatia |  | 13 | n.a. |  |
| 90 | White | Yes | Slavonija | Croatia | Slavonija i hrvatsko Podunavlje | Graševina | 2020 | Croatia |  | 13.5 | n.a. | Mitrovac position |
| 91 | Red | Yes | Plešivica | Croatia | Bregovita Hrvatska | Pinot Noir | 2018 | Croatia |  | 14 | Aging and care of wine in oak barrels | Limited production/2000 bottles |
| 92 | White | Yes | Hrvatska Istra | Croatia | Istra i Kvarner | Malvazija | 2021 | Croatia |  | 13.5 | n.a. |  |
| 93 | Red | Yes | Srednja i Južna Dalmacija | Croatia | Dalmacija | Plavac mali | 2020 | Croatia |  | 13 | n.a. | BIO |
| 94 | White | Yes | Slavonija | Croatia | Slavonija i hrvatsko Podunavlje | Graševina | 2021 | Croatia |  | 13 | The wine was fermented and aged in stainless steel tanks. 33% of the wine was fermented with natural yeasts and aged on lees -sur lie. | The grapes were cold macerated for 18-24 hours. |
| 95 | White | Yes | Plešivica | Croatia | Bregovita Hrvatska | Riesling | 2019 | Croatia |  | 13.5 | n.a. |  |
| 96 | White | Yes | Hrvatska Istra | Croatia | Istra i Kvarner | Malvazija | 2021 | Croatia |  | 13 | n.a. |  |
| 97 | Red | Yes | Srednja i Južna Dalmacija | Croatia | Dalmacija | Plavac mali | 2019 | Croatia |  | 14 | n.a. |  |
| 98 | White | Yes | Slavonija | Croatia | Slavonija i hrvatsko Podunavlje | Graševina | 2020 | Croatia |  | 13 |  | BIO |
| 99 | Red | Yes | Plešivica | Croatia | Bregovita Hrvatska | Pinot Noir | 2018 | Croatia |  | 13 | n.a. |  |
| 100 | White | Yes | Hrvatska Istra | Croatia | Istra i Kvarner | Malvazija | 2021 | Croatia |  | 12.5 | n.a. |  |
| 101 | Red | Yes | Srednja i Južna Dalmacija | Croatia | Dalmacija | Plavac mali | 2016 | Croatia | Maior Rizerva | 13.5 | n.a. | Decanter Bronze 2020 |
| 102 | White | Yes | Slavonija | Croatia | Slavonija i hrvatsko Podunavlje | Graševina | 2021 | Croatia |  | 13 | Pomale position |  |
| 103 | White | Yes | Plešivica | Croatia | Bregovita Hrvatska | Riesling | 2017 | Croatia |  | 12.5 | n.a. | The wine was macerated for 42 days by cold fermentation on its own yeasts. Limited edition |
| 104 | White | Yes | Hrvatska Istra | Croatia | Istra i Kvarner | Malvazija | 2021 | Croatia |  | 13 | n.a. |  |
| 105 | Red | Yes | Srednja i Južna Dalmacija | Croatia | Dalmacija | Plavac mali | 2018 | Croatia |  | 13.5 | Barrique |  |
| 106 | White | Yes | Slavonija | Croatia | Slavonija i hrvatsko Podunavlje | Graševina | 2020 | Croatia |  | 13 | n.a. |  |
| 107 | Red | Yes | Plešivica | Croatia | Bregovita Hrvatska | Pinot Noir | 2019 | Croatia |  | 13.5 | n.a. |  |
| 108 | White | Yes | Hrvatska Istra | Croatia | Istra i Kvarner | Malvazija | 2020 | Croatia |  | 13 | n.a. |  |
| 109 | Red | Yes | Srednja i Južna Dalmacija | Croatia | Dalmacija | Plavac mali | 2017 | Croatia |  | 13.5 | n.a. |  |
| 110 | White | Yes | Slavonija | Croatia | Slavonija i hrvatsko Podunavlje | Graševina | 2019 | Croatia |  | 12.5 | n.a. | Late harvest. Couldn't be dried completely, remained as honey texture. No water added. |
| 111 | White | Yes | Plešivica | Croatia | Bregovita Hrvatska | Riesling | 2018 | Croatia |  | 12.5 | n.a. |  |
| 112 | Red | Yes | Chianti  Classico | Italy | Toscana | n.a. | 2020 | Austria |  | 13 | n.a. |  |
| 113 | Red | Yes | Chianti  Classico | Italy | Toscana | n.a. | 2017 | Austria | Riserva | 13.5 | n.a. |  |
| 114 | White | No | Castilla y León (PGI) | Spain | Castilla  y León | Godello | 2021 | Belgium |  | 13.5 | n.a. |  |

n.a.: Information not available
