# Supplementary material for: Use of energy dispersive X-ray fluorescence to authenticate European wines with protected designation of origin. Challenges of a successful control system based on modelling
Source: Food Chem. 2025 Feb 15;465:141989. doi: 10.1016/j.foodchem.2024.141989 (PMC11649527; doi:10.1016/j.foodchem.2024.141989)
Supplement: Supplementary file 2 — Supplementary material 2 [file mmc2.docx]

**Supplementary 2:** Mass fractions (raw data) of the elements analysed in the wine for which all or some samples had concentrations higher than the LOQ of the method. All concentrations expressed in mg kg^-1^.

| **ID** | **Mg** | **P** | **Cl** | **S** | **K** | **Ca** | **Cr** | **Mn** | **Fe** | **Ni** | **Cu** | **Zn** | **As** | **Br** | **Rb** | **Sr** | **Ba** |
| --- | --- | --- | --- | --- | --- | --- | --- | --- | --- | --- | --- | --- | --- | --- | --- | --- | --- |
| **LOQ** | 1450 | 171 | 78 | 700 | 566 | 118 | 1.89 | 2.55 | 4.6 | 0.16 | 1.2 | 5.8 | 1.01 | 1.7 | 4.2 | 1.19 | 2.4 |
| **U(k=2)(%)** | 13 | 6 | 2 | 10 | 3 | 3.5 | 20 | 11 | 6.5 | 25 | 10.5 | 6.5 | 15 | 22 | 5 | 8 | 18 |
| 1 | 65.17503 | 100.7502 | 41.97507 | 106.7704 | 2267.758 | 69.63937 | 0.06911 | 0.337025 | 1.414517 | 0.012566 | 0.124758 | 0.641738 | 0.03635 | 1.594473 | 1.513695 | 1.830974 | 0.188483 |
| 2 | 56.9782 | 106.8626 | 26.85535 | 125.0701 | 1879.416 | 57.06581 | 0.064077 | 0.552285 | 1.250596 | 0.013949 | 0.136873 | 0.56667 | 0.033564 | 2.228319 | 1.586675 | 1.5457 | 0.187437 |
| 3 | 26.71017 | 188.3259 | 33.48954 | 109.8128 | 1408.982 | 87.577 | 0.04428 | 0.647066 | 1.301234 | 0.015874 | 0.13618 | 0.929453 | 0.032165 | 2.994717 | 3.916233 | 0.527177 | 0.231006 |
| 4 | 36.12512 | 100.2519 | 28.16237 | 97.18693 | 1284.074 | 101.3839 | 0.049472 | 0.718027 | 1.881758 | 0.017247 | 0.111199 | 1.235896 | 0.033133 | 1.998403 | 4.659461 | 0.579142 | 0.22966 |
| 5 | 40.61509 | 146.711 | 16.28963 | 159.9842 | 1466.047 | 78.60103 | 0.062449 | 0.919975 | 1.924193 | 0.013831 | 0.097236 | 1.483276 | 0.031015 | 2.11657 | 2.4175 | 1.264493 | 0.178127 |
| 6 | 57.96714 | 135.2718 | 50.60999 | 115.8931 | 1730.167 | 90.90436 | 0.055059 | 1.712911 | 0.87965 | 0.026012 | 0.185988 | 0.426168 | 0.032949 | 1.744993 | 2.694008 | 0.510708 | 0.427035 |
| 7 | 29.71133 | 151.8433 | 24.92353 | 125.1764 | 1123.957 | 92.49155 | 0.074348 | 1.354365 | 3.645365 | 0.030276 | 0.044072 | 1.306077 | 0.02721 | 2.639364 | 3.54189 | 0.859221 | 0.226111 |
| 8 | 60.38902 | 118.1019 | 23.99068 | 195.69 | 1677.519 | 73.97489 | 0.05374 | 0.611012 | 0.831392 | 0.007226 | 0.097997 | 0.433082 | 0.031612 | 1.31415 | 2.169928 | 0.757781 | 0.232121 |
| 9 | 52.63849 | 149.9672 | 32.05382 | 198.9683 | 1578.38 | 107.7979 | 0.064395 | 0.71923 | 2.131389 | 0.031291 | 0.126976 | 0.780451 | 0.030384 | 2.336365 | 3.422466 | 0.87659 | 0.17686 |
| 10 | 32.27516 | 183.5066 | 45.46759 | 102.9034 | 1552.161 | 93.76643 | 0.063651 | 1.16294 | 2.588139 | 0.011486 | 0.275181 | 0.613056 | 0.041158 | 2.092813 | 2.269886 | 1.000224 | 0.304374 |
| 12 | 0.418587 | 302.0098 | 46.47833 | 330.2239 | 1668.004 | 169.659 | 0.068263 | 1.555361 | 3.709449 | 0.049775 | 0.180139 | 1.799497 | 0.036976 | 2.58737 | 3.078487 | 0.630014 | 0.198627 |
| 13 | 39.35405 | 137.9884 | 35.6545 | 129.3354 | 1262.678 | 111.819 | 0.092511 | 0.856183 | 3.380745 | 0.020407 | 0.220848 | 1.022613 | 0.032198 | 4.004289 | 4.01608 | 0.881579 | 0.138314 |
| 14 | 25.69278 | 123.3704 | 21.77063 | 217.1646 | 1574.174 | 83.63154 | 0.042013 | 0.682153 | 1.376956 | 0.008583 | 0.100742 | 0.520876 | 0.034785 | 2.057754 | 1.997218 | 2.119193 | 0.170312 |
| 15 | 39.88411 | 108.024 | 26.58192 | 259.9159 | 1618.048 | 99.12748 | 0.043625 | 0.691221 | 2.25875 | 0.011012 | 0.062261 | 0.500203 | 0.030919 | 2.622572 | 1.652661 | 1.739911 | 0.245654 |
| 16 | 13.03415 | 160.5644 | 38.99612 | 132.1971 | 1252.788 | 100.5275 | 0.064532 | 0.79203 | 2.839888 | 0.011142 | 0.16992 | 0.480511 | 0.03482 | 3.261438 | 1.725196 | 1.878867 | 0.297592 |
| 17 | 0.467998 | 210.6057 | 24.93589 | 293.9148 | 1321.523 | 146.1266 | 0.069837 | 1.132357 | 4.774764 | 0.030837 | 0.094325 | 1.647518 | 0.036732 | 4.657764 | 3.880034 | 0.885206 | 0.289778 |
| 18 | 20.82824 | 160.1457 | 24.64267 | 103.6157 | 1303.754 | 76.28945 | 0.09546 | 1.000344 | 2.837954 | 0.050117 | 0.117337 | 1.139159 | 0.029434 | 3.311278 | 4.245993 | 0.724702 | 0.310246 |
| 19 | 34.24053 | 147.1668 | 32.45624 | 338.6142 | 1707.162 | 138.5483 | 0.059687 | 0.709011 | 1.312213 | 0.012209 | 0.094505 | 0.661985 | 0.034818 | 1.882406 | 1.515692 | 2.080911 | 0.151931 |
| 20 | 5.948325 | 156.421 | 9.84706 | 203.9637 | 1012.994 | 65.81702 | 0.067086 | 0.628986 | 2.110171 | 0.021956 | 0.101239 | 0.814795 | 0.030494 | 2.210598 | 2.565546 | 0.694039 | 0.173205 |
| 21 | 34.58609 | 173.0411 | 28.97872 | 136.8433 | 1494.497 | 92.90949 | 0.118814 | 0.858904 | 2.571724 | 0.019953 | 0.236267 | 0.841672 | 0.033558 | 4.150312 | 1.677448 | 1.637088 | 0.193639 |
| 22 | 31.08062 | 219.986 | 28.54609 | 165.8613 | 1472.641 | 79.40406 | 0.042277 | 0.793546 | 1.658541 | 0.015643 | 0.272689 | 0.38261 | 0.033399 | 2.454623 | 2.805525 | 1.133456 | 0.22872 |
| 23 | 47.93402 | 194.9109 | 50.56614 | 208.0051 | 1791.923 | 100.3118 | 0.084656 | 1.564533 | 2.693432 | 0.018304 | 0.105706 | 0.995279 | 0.032947 | 3.367476 | 1.783266 | 1.349919 | 0.333133 |
| 24 | 37.6766 | 195.5437 | 33.70417 | 193.4219 | 1308.942 | 126.5 | 0.076045 | 1.05582 | 3.881542 | 0.031067 | 0.093202 | 1.258452 | 0.035704 | 3.936257 | 4.067945 | 1.153195 | 0.333393 |
| 25 | 43.02049 | 170.2435 | 27.42689 | 149.2983 | 1538.146 | 111.4231 | 0.063488 | 1.220787 | 4.249626 | 0.034465 | 0.126523 | 1.164554 | 0.032651 | 4.279103 | 5.035519 | 0.89473 | 0.242616 |
| 26 | 12.7012 | 188.5056 | 25.28726 | 365.4752 | 1667.919 | 93.15804 | 0.058791 | 0.776933 | 1.349732 | 0.020005 | 0.283338 | 0.366624 | 0.032253 | 1.456698 | 2.673743 | 1.084766 | 0.198418 |
| 27 | 7.372179 | 189.6896 | 14.90982 | 170.7204 | 1335.558 | 81.40905 | 0.106017 | 0.999735 | 0.945735 | 0.026752 | 0.10354 | 1.15678 | 0.03666 | 2.519154 | 1.985599 | 1.730958 | 0.153577 |
| 28 | 5.493414 | 168.2932 | 40.47262 | 215.9568 | 1252.678 | 118.6798 | 0.073262 | 1.825905 | 3.137771 | 0.024152 | 0.288619 | 1.139178 | 0.02858 | 4.1421 | 2.878538 | 0.960049 | 0.214149 |
| 29 | 7.524783 | 217.4058 | 34.10266 | 395.9335 | 1920.373 | 125.3174 | 0.080834 | 0.860105 | 2.229733 | 0.011353 | 0.282917 | 0.721144 | 0.037238 | 2.282866 | 1.448191 | 2.323282 | 0.185735 |
| 30 | 24.19992 | 185.3304 | 39.77328 | 98.22197 | 1508.112 | 101.5045 | 0.056143 | 1.416895 | 2.057762 | 0.035765 | 0.173837 | 1.019316 | 0.03327 | 4.496468 | 4.93314 | 0.818031 | 0.289867 |
| 31 | 52.07234 | 131.2575 | 35.08207 | 234.0561 | 1601.402 | 91.56776 | 0.066312 | 0.750974 | 1.970357 | 0.019007 | 0.100524 | 0.630599 | 0.029988 | 3.726573 | 1.722848 | 1.817037 | 0.184998 |
| 32 | 26.12752 | 161.2679 | 62.88179 | 173.9716 | 1498.689 | 96.24702 | 0.064676 | 2.401217 | 1.062848 | 0.025439 | 0.1065 | 0.390214 | 0.034063 | 5.977387 | 2.037736 | 0.620893 | 0.333299 |
| 33 | 18.99189 | 176.5572 | 25.82807 | 151.7678 | 1290.181 | 118.4833 | 0.061221 | 1.039077 | 4.66201 | 0.034804 | 0.538827 | 1.263414 | 0.03061 | 4.524473 | 3.643899 | 0.936763 | 0.22224 |
| 34 | 23.56065 | 193.8588 | 23.48465 | 258.5647 | 1465.875 | 149.8378 | 0.084581 | 0.90205 | 2.422784 | 0.026619 | 0.063113 | 2.715597 | 0.031771 | 6.281722 | 4.386601 | 0.87586 | 0.145118 |
| 35 | 10.94528 | 225.5434 | 27.08572 | 632.9351 | 2373.755 | 97.67729 | 0.073787 | 0.834195 | 2.062071 | 0.017674 | 0.267314 | 0.565114 | 0.036673 | 3.234716 | 2.543677 | 1.356009 | 0.26643 |
| 36 | 22.4619 | 198.6849 | 28.49378 | 399.7629 | 1466.312 | 210.4154 | 0.082425 | 1.234121 | 4.100074 | 0.019818 | 0.095937 | 1.453921 | 0.033781 | 5.190965 | 4.07305 | 1.126473 | 0.333753 |
| 37 | 50.29473 | 138.317 | 29.58312 | 254.4548 | 1685.091 | 131.7013 | 0.066374 | 1.061978 | 2.028083 | 0.011279 | 0.072881 | 0.793013 | 0.031668 | 6.412648 | 2.505713 | 1.78775 | 0.209966 |
| 38 | 57.89196 | 121.4523 | 26.9293 | 344.6403 | 2388.706 | 82.12528 | 0.070761 | 0.601908 | 0.983573 | 0.006634 | 0.101276 | 0.419257 | 0.032285 | 4.737427 | 2.502274 | 0.965883 | 0.219358 |
| 39 | 56.25885 | 108.005 | 28.23077 | 266.3302 | 1976.443 | 118.9337 | 0.055089 | 0.693448 | 1.420117 | 0.015139 | 0.076536 | 0.579485 | 0.031539 | 2.703564 | 1.921385 | 2.504234 | 0.160221 |
| 41 | 41.82844 | 138.7456 | 31.04989 | 152.2586 | 1811.443 | 82.53587 | 0.066263 | 0.698933 | 1.955652 | 0.009077 | 0.105294 | 0.736149 | 0.032224 | 4.876196 | 1.886666 | 1.99196 | 0.226473 |
| 42 | 78.47403 | 128.7137 | 30.92911 | 282.4235 | 2252.399 | 107.9695 | 0.090516 | 0.936274 | 1.545843 | 0.0165 | 0.116445 | 0.56761 | 0.034415 | 4.534755 | 2.750836 | 0.729313 | 0.371493 |
| 43 | 26.53605 | 162.2961 | 15.16505 | 200.5419 | 1180.613 | 107.8256 | 0.096664 | 0.698488 | 1.424861 | 0.028348 | 0.303933 | 1.384894 | 0.035784 | 6.755772 | 4.124474 | 0.967102 | 0.314622 |
| 44 | n.d. | 144.8667 | 8.465399 | 219.7601 | 946.548 | 78.07154 | 0.097827 | 0.585258 | 1.3096 | 0.016163 | 0.158649 | 0.953596 | 0.031049 | 4.154651 | 3.355451 | 0.597593 | 0.342818 |
| 45 | 19.97078 | 151.9173 | 27.81581 | 434.4078 | 1717.513 | 185.0855 | 0.074247 | 0.854061 | 2.329454 | 0.011289 | 0.084234 | 0.777208 | 0.032565 | 4.923333 | 1.762396 | 2.085871 | 0.199295 |
| 46 | 47.22702 | 94.38224 | 52.234 | 159.9585 | 1540.884 | 106.8044 | 0.078635 | 0.596599 | 1.487223 | 0.012394 | 0.052566 | 0.508989 | 0.03248 | 6.442496 | 1.257302 | 1.827831 | 0.161971 |
| 47 | 9.82555 | 186.8354 | 26.52791 | 106.8109 | 1300.55 | 105.0987 | 0.103835 | 0.873334 | 2.530817 | 0.02542 | 0.161138 | 1.974158 | 0.034899 | 5.609676 | 4.288688 | 0.881521 | 0.199053 |
| 48 | n.d | 179.6093 | 31.1695 | 227.7229 | 1219.109 | 120.2757 | 0.103852 | 1.060997 | 4.152276 | 0.029672 | 0.175784 | 1.538446 | 0.035966 | 6.574138 | 4.45484 | 1.021884 | 0.267947 |
| 49 | 28.59216 | 132.6615 | 31.14003 | 337.017 | 1722.336 | 95.01961 | 0.086053 | 0.779355 | 1.69666 | 0.015969 | 0.20493 | 0.782016 | 0.037704 | 4.821619 | 1.587985 | 2.608642 | 0.176985 |
| 51 | 42.85492 | 153.822 | 22.69369 | 128.2052 | 1218.827 | 110.3924 | 0.071576 | 0.714518 | 2.190305 | 0.020687 | 0.112535 | 0.887872 | 0.032685 | 5.46335 | 5.182425 | 0.710794 | 0.228795 |
| 52 | 30.98595 | 106.604 | 31.29346 | 194.7423 | 1675.031 | 58.42435 | 0.072499 | 0.413814 | 0.840254 | 0.01507 | 0.445583 | 0.369011 | 0.030955 | 2.31467 | 2.232804 | 0.187357 | 0.131964 |
| 53 | 17.4787 | 180.293 | 39.64257 | 174.7695 | 1287.564 | 90.13285 | 0.099419 | 1.488504 | 1.935656 | 0.048091 | 0.10728 | 3.137002 | 0.033294 | 7.644592 | 2.668579 | 1.746992 | 0.351895 |
| 54 | 17.56121 | 111.8444 | 17.15092 | 121.7693 | 1002.535 | 98.73622 | 0.062265 | 1.093555 | 1.148398 | 0.038761 | 0.092779 | 0.342251 | 0.031751 | 3.520241 | 5.801367 | 0.175249 | 0.189269 |
| 55 | 36.73408 | 133.5078 | 22.45898 | 246.2062 | 1450.689 | 111.2239 | 0.084699 | 0.592449 | 1.223036 | 0.023059 | 0.103767 | 0.569833 | 0.031042 | 2.219468 | 2.153394 | 0.171172 | 0.141461 |
| 56 | 40.64455 | 155.8636 | 21.66856 | 564.5692 | 2090.24 | 78.32573 | 0.056044 | 0.478215 | 1.496415 | 0.009818 | 0.154632 | 0.283492 | 0.03109 | 3.154825 | 1.568413 | 0.210676 | 0.139496 |
| 57 | 19.79197 | 122.1046 | 12.10547 | 56.27544 | 895.7251 | 95.2182 | 0.063927 | 0.866443 | 0.330291 | 0.049468 | 0.089042 | 0.62938 | 0.027778 | 5.135494 | 3.901088 | 0.235542 | 0.197109 |
| 58 | 12.19791 | 131.0587 | 12.68576 | 72.8944 | 827.9682 | 91.51761 | 0.079255 | 0.750922 | 0.693979 | 0.030969 | 0.131203 | 0.694978 | 0.025974 | 5.933784 | 4.663044 | 0.347655 | 0.216452 |
| 59 | 35.54266 | 153.3309 | 23.91746 | 245.5882 | 1851.024 | 74.25348 | 0.046415 | 1.211318 | 1.419981 | 0.012733 | 0.12487 | 0.374198 | 0.03286 | 3.172264 | 2.168378 | 0.45676 | 0.307656 |
| 60 | 12.45388 | 178.858 | 18.38552 | 63.67354 | 967.1105 | 97.75664 | 0.055481 | 0.636419 | 0.718673 | 0.02516 | 0.063868 | 0.77867 | 0.021289 | 6.734174 | 4.323651 | 0.45417 | 0.15096 |
| 61 | 113.5066 | 99.24845 | 31.92413 | 66.17825 | 2378.865 | 75.14288 | 0.040066 | 1.151784 | 1.702139 | 0.017171 | 0.107429 | 0.370279 | 0.036984 | 3.270872 | 2.765866 | 0.431038 | 0.437202 |
| 62 | 65.96803 | 73.47817 | 37.94572 | 101.9418 | 1859.002 | 62.53031 | 0.053366 | 0.150912 | 0.642577 | 0.009623 | 0.065614 | 0.33288 | 0.032807 | 1.678399 | 2.956116 | 1.731327 | 0.127291 |
| 63 | 28.68522 | 145.2358 | 18.10384 | 65.28122 | 1107.709 | 122.6916 | 0.058399 | 0.626955 | 0.845114 | 0.028193 | 0.06142 | 0.558151 | 0.024837 | 5.304619 | 2.459826 | 0.407789 | 0.541034 |
| 64 | 28.39945 | 119.2682 | 22.94494 | 71.30099 | 1059.643 | 89.0974 | 0.056293 | 0.925893 | 0.477969 | 0.023139 | 0.0632 | 0.589518 | 0.025902 | 4.557975 | 3.485653 | 0.282154 | 0.242438 |
| 65 | 42.41726 | 100.1731 | 27.36517 | 76.42011 | 1196.021 | 90.46783 | 0.064364 | 1.835803 | 1.201353 | 0.024756 | 0.040316 | 1.685147 | 0.023341 | 5.151281 | 3.529084 | 0.677949 | 0.394321 |
| 66 | 33.78871 | 121.3508 | 36.34847 | 84.79142 | 1361.217 | 88.12016 | 0.071177 | 1.545814 | 6.494604 | 0.037554 | 0.32139 | 0.622692 | 0.030567 | 4.31125 | 4.094225 | 0.365057 | 0.422697 |
| 67 | 63.03375 | 132.945 | 24.3368 | 96.9942 | 1914.277 | 72.27001 | 0.04449 | 2.020931 | 1.435008 | 0.042391 | 0.095695 | 0.476797 | 0.032738 | 2.866658 | 1.636891 | 0.319404 | 0.431887 |
| 68 | 34.56214 | 78.34213 | 12.42439 | 66.52814 | 1066.204 | 72.18906 | 0.052071 | 1.103898 | 0.199212 | 0.031242 | 0.047703 | 0.546238 | 0.026875 | 5.144244 | 3.947963 | 0.284205 | 0.278494 |
| 69 | 54.49241 | 111.7583 | 35.06383 | 132.7704 | 1606.96 | 64.85033 | 0.066636 | 0.421431 | 0.904995 | 0.016209 | 0.081044 | 0.411075 | 0.032868 | 2.758209 | 3.751903 | 0.325078 | 0.135074 |
| 70 | 5.243468 | 185.8515 | 11.17948 | 121.4033 | 721.0857 | 107.3917 | 0.046917 | 0.929231 | 1.345179 | 0.034662 | 0.465666 | 0.747166 | 0.02801 | 7.614513 | 3.237253 | 0.327017 | 0.293054 |
| 71 | 14.93803 | 92.27892 | 6.465367 | 292.346 | 858.374 | 165.4869 | 0.033751 | 0.528407 | 0.899671 | 0.011976 | 0.058793 | 0.566514 | 0.027582 | 6.082127 | 0.709503 | 0.183999 | 0.098351 |
| 72 | 16.68136 | 160.1449 | 12.89888 | 79.27622 | 911.8452 | 93.7784 | 0.050166 | 1.41144 | 1.102645 | 0.027456 | 0.131517 | 0.616234 | 0.023049 | 8.159168 | 4.0384 | 0.337268 | 0.209479 |
| 73 | 28.00293 | 119.0759 | 16.12336 | 108.0595 | 1191.088 | 114.4106 | 0.033048 | 0.869948 | 1.110682 | 0.023004 | 0.047304 | 0.638286 | 0.023652 | 9.997435 | 3.839111 | 0.296787 | 0.185978 |
| 74 | 39.17856 | 164.8614 | 26.69483 | 282.7961 | 1816.592 | 131.0796 | 0.068046 | 2.632655 | 1.969204 | 0.029885 | 0.062069 | 0.533795 | 0.033104 | 5.497035 | 3.034496 | 0.60552 | 0.502071 |
| 75 | 11.42025 | 136.4402 | 10.90693 | 105.7106 | 938.601 | 124.0185 | 0.020685 | 0.859239 | 0.550868 | 0.059192 | 0.248225 | 0.814368 | 0.022595 | 9.639393 | 3.698867 | 0.363108 | 0.186487 |
| 76 | 19.75196 | 109.6848 | 12.83075 | 139.2028 | 902.4855 | 102.0394 | 0.047055 | 1.817084 | 0.885867 | 0.017731 | 0.047737 | 0.681961 | 0.02421 | 5.243595 | 4.051187 | 0.26085 | 0.288128 |
| 77 | 85.79764 | 141.0643 | 27.9396 | 266.5298 | 2232.747 | 112.5798 | 0.084261 | 0.631184 | 0.957891 | 0.012407 | 0.098219 | 0.687014 | 0.03205 | 5.982035 | 3.010661 | 0.285351 | 0.146294 |
| 78 | 29.88784 | 84.20733 | 18.90863 | 40.25502 | 1169.566 | 77.45476 | 0.04374 | 1.121065 | 0.676083 | 0.022387 | 0.071982 | 0.914073 | 0.023765 | 7.152775 | 4.381281 | 0.514209 | 0.308594 |
| 79 | 7.086551 | 116.3048 | 12.02182 | 93.63955 | 1093.349 | 99.28295 | 0.033675 | 0.632693 | 0.578296 | 0.027199 | 0.670577 | 0.736307 | 0.024285 | 9.810628 | 1.958305 | 0.424494 | 0.217266 |
| 80 | 26.73 | 178.4165 | 15.82229 | 397.6375 | 1732.933 | 231.124 | 0.056605 | 0.772729 | 1.648137 | 0.023256 | 0.105751 | 0.515152 | 0.034226 | 6.559198 | 7.739573 | 0.615199 | 0.183858 |
| 81 | 36.74772 | 200.7019 | 14.14313 | 80.52734 | 1388.681 | 85.17023 | 0.054287 | 1.223191 | 0.717282 | 0.046587 | 0.046587 | 0.585607 | 0.026951 | 6.719274 | 4.978239 | 0.830091 | 0.39695 |
| 82 | 93.38939 | 139.3726 | 27.96611 | 127.396 | 2137.891 | 73.50695 | 0.025464 | 2.321517 | 2.134639 | 0.031074 | 0.114803 | 0.431157 | 0.031506 | 5.816094 | 2.365539 | 0.552002 | 0.316355 |
| 83 | 50.63498 | 159.5853 | 28.06467 | 231.9101 | 1929.746 | 103.9936 | 0.076938 | 0.551551 | 1.456537 | 0.015388 | 0.095692 | 0.453936 | 0.036065 | 3.507903 | 2.552426 | 0.262071 | 0.164936 |
| 84 | 13.87162 | 127.3917 | 12.97678 | 57.6573 | 966.4278 | 86.17561 | 0.03704 | 1.422056 | 1.032964 | 0.010385 | 0.053656 | 0.595754 | 0.027693 | 9.352059 | 3.270244 | 0.279357 | 0.264818 |
| 85 | 15.771 | 140.8374 | 10.70529 | 79.1472 | 808.2391 | 94.97722 | 0.050041 | 0.640591 | 0.489826 | 0.017964 | 0.060306 | 0.546925 | 0.024379 | 9.781449 | 3.292454 | 0.200165 | 0.168408 |
| 86 | 22.64722 | 87.48272 | 15.93216 | 121.2175 | 1011.244 | 98.06253 | 0.056021 | 2.461367 | 0.777875 | 0.028189 | 0.128456 | 1.112218 | 0.025334 | 3.925413 | 2.132376 | 0.546296 | 0.142372 |
| 87 | 74.41203 | 106.5332 | 23.98356 | 183.7406 | 1836.143 | 101.0821 | 0.074465 | 0.592599 | 0.58279 | 0.013377 | 0.075357 | 0.581006 | 0.032551 | 3.426286 | 5.317788 | 0.244798 | 0.13154 |
| 88 | 20.60617 | 125.6329 | 32.65505 | 72.32764 | 673.0109 | 107.5984 | 0.065521 | 1.655701 | 0.676413 | 0.027943 | 0.301913 | 0.542158 | 0.02184 | 0.691187 | 3.85067 | 0.346236 | 0.267546 |
| 89 | 20.23625 | 108.8944 | 16.13197 | 227.924 | 995.3895 | 83.33579 | 0.057449 | 0.765572 | 1.210178 | 0.012073 | 0.147786 | 0.441692 | 0.028725 | 0.539939 | 2.255087 | 0.268096 | 0.114482 |
| 90 | 21.92403 | 147.5485 | 11.61325 | 65.66594 | 991.2839 | 95.44339 | 0.04138 | 1.045358 | 0.338503 | 0.050199 | 0.065801 | 0.570504 | 0.024082 | 0.454843 | 3.757456 | 0.261848 | 0.184515 |
| 91 | 9.779047 | 154.2822 | 17.58964 | 318.3514 | 953.4719 | 110.5935 | 0.050432 | 0.667627 | 2.73975 | 0.044028 | 0.148495 | 0.558757 | 0.026017 | 0.412664 | 3.696762 | 0.804114 | 0.140089 |
| 92 | 33.91414 | 103.0109 | 17.57212 | 250.4381 | 1194.905 | 158.5567 | 0.046069 | 1.449574 | 1.013838 | 0.038071 | 0.12445 | 1.168681 | 0.023354 | 0.499081 | 5.546515 | 0.287291 | 0.192274 |
| 93 | 60.79402 | 145.9378 | 34.53386 | 136.319 | 1576.017 | 96.88131 | 0.064823 | 0.613964 | 0.769539 | 0.011575 | 0.109273 | 0.601463 | 0.035189 | 0.68851 | 3.299478 | 0.345876 | 0.105568 |
| 94 | n.d. | 151.2746 | 10.3585 | 86.48201 | 728.2426 | 151.2509 | 0.052267 | 1.089699 | 0.475217 | 0.040576 | 0.06946 | 1.051187 | 0.026134 | 0.938056 | 2.215848 | 0.367245 | 0.182247 |
| 95 | n.d. | 143.4681 | 5.169932 | 276.8034 | 618.8492 | 256.9247 | 0.061428 | 0.29644 | 0.483484 | 0.017255 | 0.123546 | 0.578386 | 0.027953 | 0.632912 | 2.828088 | 0.319562 | 0.137695 |
| 96 | n.d. | 162.6728 | 10.40843 | 122.5477 | 697.5734 | 116.5941 | 0.035152 | 1.261224 | 0.70954 | 0.020505 | 0.068676 | 0.933143 | 0.025062 | 0.87 | 3.791809 | 0.312784 | 0.218395 |
| 97 | 49.28987 | 120.5667 | 46.84009 | 108.0365 | 1340.522 | 87.9451 | 0.062837 | 0.477129 | 1.747305 | 0.017334 | 0.126108 | 2.058024 | 0.031202 | 0.839417 | 3.175658 | 0.197612 | 0.136075 |
| 98 | 22.50485 | 167.1418 | 13.99988 | 78.57061 | 1071.297 | 91.8996 | 0.076602 | 1.371922 | 5.921867 | 0.043638 | 0.046463 | 0.418169 | 0.021976 | 0.800235 | 3.207534 | 0.448935 | 0.237967 |
| 99 | 45.66314 | 119.3926 | 15.66742 | 66.76991 | 1457.396 | 71.10904 | 0.043209 | 0.197834 | 0.68849 | 0.009999 | 0.059636 | 0.226045 | 0.027854 | 0.687775 | 2.8843 | 0.418522 | 0.124985 |
| 100 | 19.78325 | 146.7978 | 21.27855 | 121.6854 | 1154.76 | 126.7753 | 0.04079 | 1.062585 | 0.87291 | 0.028553 | 0.077501 | 0.773314 | 0.022435 | 1.090458 | 4.33294 | 0.389546 | 0.212789 |
| 101 | 47.55044 | 143.3187 | 35.83894 | 257.646 | 1728.435 | 106.3182 | 0.079479 | 0.564249 | 2.196276 | 0.023695 | 0.530187 | 1.337314 | 0.040973 | 0.97892 | 3.933945 | 0.369748 | 0.153527 |
| 102 | 15.88072 | 127.0293 | 9.50498 | 54.27044 | 781.1785 | 106.6725 | 0.055504 | 0.916966 | 0.326784 | 0.058788 | 0.042367 | 0.635504 | 0.024632 | 1.017136 | 4.418316 | 0.278505 | 0.205923 |
| 103 | 12.43973 | 125.951 | 10.96167 | 238.7832 | 921.3123 | 199.8002 | 0.038159 | 0.412992 | 0.374158 | 0.025664 | 0.069564 | 0.520039 | 0.023638 | 0.75642 | 3.577123 | 0.788838 | 0.178299 |
| 104 | 7.111503 | 170.4225 | 14.57616 | 57.88507 | 928.5831 | 99.83429 | 0.061223 | 0.706308 | 0.843972 | 0.042544 | 0.070562 | 0.834979 | 0.022829 | 1.174643 | 4.754261 | 0.571065 | 0.253883 |
| 105 | 40.84843 | 187.1297 | 40.02001 | 195.5294 | 1697.89 | 105.6182 | 0.089817 | 0.729044 | 1.296131 | 0.007644 | 0.101283 | 0.745287 | 0.039653 | 0.928265 | 3.230533 | 0.213076 | 0.14428 |
| 106 | 24.88813 | 118.4295 | 12.65317 | 95.38952 | 1093.596 | 92.35849 | 0.055414 | 0.744427 | 0.280554 | 0.036943 | 0.048444 | 0.689362 | 0.027184 | 1.45958 | 4.287776 | 0.281251 | 0.164499 |
| 107 | 35.10434 | 125.563 | 14.73743 | 95.28651 | 1368.815 | 79.71705 | 0.050237 | 0.245685 | 0.278321 | 0.011734 | 0.053904 | 0.342492 | 0.028235 | 0.661149 | 2.230599 | 0.277954 | 0.124309 |
| 108 | 23.11838 | 152.1225 | 15.64299 | 112.4619 | 1116.424 | 97.0302 | 0.038357 | 0.56932 | 2.350444 | 0.024151 | 0.221264 | 0.649586 | 0.027347 | 1.514044 | 1.832621 | 0.420864 | 0.170121 |
| 109 | 74.52432 | 88.94513 | 21.82805 | 126.2723 | 1561.139 | 76.91861 | 0.047171 | 0.346071 | 0.532994 | 0.006613 | 0.054225 | 0.281706 | 0.034828 | 0.611466 | 2.761515 | 0.171933 | 0.130052 |
| 110 | n.d. | 151.7149 | 11.35128 | 41.72471 | 1130.834 | 92.88684 | 0.124878 | 0.74085 | 0.296761 | 0.030869 | 0.039287 | 0.586506 | 0.050512 | 1.508359 | 1.932102 | 0.2245 | 0.260981 |
| 111 | 31.80306 | 98.41948 | 11.51762 | 68.25732 | 1152.815 | 63.83209 | 0.056817 | 0.234368 | 0.127514 | 0.011622 | 0.040353 | 0.46002 | 0.019692 | 0.904868 | 12.91705 | 0.685349 | 0.182394 |
| 112 | 32.58476 | 192.3021 | 31.51737 | 110.9128 | 1682.287 | 86.35475 | 0.099352 | 0.826231 | 1.607882 | 0.008916 | 0.154547 | 1.001582 | 0.030994 | 0.168983 | 4.044967 | 0.647483 | 0.215262 |
| 113 | n.d. | 232.7393 | 35.0973 | 211.7511 | 1481.953 | 116.8314 | 0.106114 | 1.244099 | 1.832759 | 0.013722 | 0.150481 | 1.367136 | 0.037049 | 0.277635 | 4.406945 | 0.901057 | 0.276263 |
| 114 | 6.61767 | 102.6195 | 14.87502 | 136.7996 | 880.229 | 119.9991 | 0.076058 | 1.00333 | 0.286168 | 0.023451 | 0.191412 | 0.875616 | 0.023134 | 0.070037 | 3.289504 | 0.437333 | 0.129298 |
