# Supplementary material for: Use of energy dispersive X-ray fluorescence to authenticate European wines with protected designation of origin. Challenges of a successful control system based on modelling
Source: Food Chem. 2025 Feb 15;465:141989. doi: 10.1016/j.foodchem.2024.141989 (PMC11649527; doi:10.1016/j.foodchem.2024.141989)
Supplement: Supplementary file 3 — Supplementary material 3: Biplots of the different PLS-DA models. [file mmc3.docx]

**Supplementary 3:** Biplots of the different PLS-DA models

a) Biplot Spanish red wines for which more than three samples were available

b) Biplot Italian red wines for which more than three samples were available

c) Biplot Croatian red wines for which more than three samples were available

d) Biplot Spanish white wines for which more than three samples were available

e) Biplot Croatian white wines for which more than three samples were available
